# Randomization in survival studies: An evaluation method that takes into account selection and chronological bias

**DOI:** 10.1371/journal.pone.0217946

**Published:** 2019-06-03

**Authors:** Marcia Viviane Rückbeil, Ralf-Dieter Hilgers, Nicole Heussen

**Affiliations:** 1 Department of Medical Statistics, RWTH Aachen University, Aachen, Germany; 2 Center for Biostatistics and Epidemiology, Sigmund Freud Private University, Vienna, Austria; NIH/NCI/DCP/BRG, UNITED STATES

## Abstract

The random allocation of patients to treatments is a crucial step in the design and conduct of a randomized controlled trial. For this purpose, a variety of randomization procedures is available. In the case of imperfect blinding, the extent to which a randomization procedure forces balanced group sizes throughout the allocation process affects the predictability of allocations. As a result, some randomization procedures perform superior with respect to selection bias, whereas others are less susceptible to chronological bias. The choice of a suitable randomization procedure therefore depends on the expected risk for selection and chronological bias within the particular study in question. To enable a sound comparison of different randomization procedures, we introduce a model for the combined effect of selection and chronological bias in randomized studies with a survival outcome. We present an evaluation method to quantify the influence of bias on the test decision of the log-rank test in a randomized parallel group trial with a survival outcome. The effect of selection and chronological bias and the dependence on the study setting are illustrated in a sensitivity analysis. We conclude with a case study to showcase the application of our model for comparing different randomization procedures in consideration of the expected type I error probability.

## Introduction

One of the main purposes of randomization is to improve comparability between treatment groups by balancing observed and unobserved covariates in expectation [1]. Randomization furthermore helps to mitigate the risk of selection bias and, depending on the randomization procedure, can protect against imbalanced group sizes throughout the allocation process [1]. Despite the many benefits of randomization, there are also some limitations; for a comprehensive discussion, see [2]: One issue that cannot be addressed by randomization is that patients usually enter a clinical trial sequentially and are often treated immediately [1, 2]. Consequently, new patients will be enrolled and assigned to therapies, while others have already received treatment [2]. This delay in time entails several potential sources of bias: On the one hand, the treatment success itself may be affected by unobserved time trends (chronological bias) [3, 4]. These may result from, for example, improved treatment performance due to experience gain [5, 6], or changes in inclusion or exclusion criteria [4, 7]. On the other hand, the sequential enrollment creates the risk for selection bias whenever blinding cannot be fully attained [8, 9].

There are many different randomization procedures for randomly assigning patients to treatments; for a detailed overview, see [1]. In general, randomization procedures that protect well against selection bias perform poorly with regard to time trends [10]. This is caused by the opposing behaviors of either forcing balanced groups or maintaining randomness: The more balance is forced by a randomization procedure, the more robust it is with regard to time trends [4] but at the same time the more susceptible it is to selection bias [11–13]. Therefore, there cannot be a universally best randomization procedure. Instead, the choice of a suitable randomization procedure always depends on the specific requirements of the study at hand [10]. In the case of two treatment groups and a normally distributed outcome, several models have been proposed to study the isolated effect of selection bias [12, 14–16] or the isolated effect of time trends [4]. Another model was introduced to compare more than two treatment groups in the presence of selection bias [17]. Recently, an approach for the combined assessment of chronological and selection bias in the case of a normally distributed outcome was published [10]. For survival outcomes, a single model has been proposed for the case where survival data follow an exponential distribution and an F-test is performed to compare two treatments [9]. This model is limited to the evaluation of selection bias only and does not account for time trends. In addition, the F-test is rarely considered in clinical trials, as it can only be used in very specific situations. Instead, methods that do not impose any distributional assumptions on the survival times are often used in practice [18–20]. Therefore, the previous evaluation method can only be applied to a very limited extent to most survival studies.

The objective of this paper is to provide a method for the evaluation and comparison of different randomization procedures that is applicable to most studies with a survival outcome and thus enable researchers to make a scientifically sound choice. We propose a semi-parametric bias model based on the hazard functions to model the simultaneous effect of selection and chronological bias. Based on this model, we derive an approximation formula that describes the impact on the distribution of the log-rank statistic [21, 22]. The relevance to consider bias when selecting a randomization procedure and the dependence on the specific scenario is shown in a sensitivity analysis. We conclude with a case study to illustrate the application of our evaluation method to compare different randomization procedures if selection bias and time trend are anticipated. The performance of the different randomization procedures is evaluated by means of the expected type I error probability.

## Prerequisites and setting

We consider a randomized, two-arm, parallel group trial where a control (C) and an experimental (E) treatment are compared with regard to a survival outcome. With an intended 1:1 allocation ratio, a total of *n* patients are enrolled over an accrual period of length *A* ≥ 0. The maximum duration of follow-up is of length *F*, with *F* > *A*, so that all patients who have not yet had an event until then are regarded as right-censored. We assume that throughout the accrual period, patients enter the trial sequentially according to a uniform distribution. Furthermore, there is a random censoring mechanism that can be modeled by a probability distribution *S*_*cen*_ which is independent of the survival distributions. Let *d* denote the number of observed events, where *d* ≤ *n*, with the distinct ordered event times *τ*_1_ < *τ*_2_ < … < *τ*_*d*_.

We assume that patients are assigned to treatments according to a randomization sequence ***z*** = (*z*_1_, …, *z_n_*) ∈ {0, 1}^*n*^ and treated immediately. Particularly, the *i*th patient who enters the trial is assigned to treatment ***z***_*i*_ ∈ {0, 1}. If *z*_*i*_ = 0, the respective patient is assigned to the control treatment, otherwise to the experimental treatment. The randomization sequence ***z*** can be interpreted as the realization of a random variable ***Z*** whose probability distribution depends on the randomization procedure used. The available randomization procedures can be classified into (I) unrestricted and (II) restricted randomization procedures. Among the restricted randomization procedures, there are (a) procedures with forced final balance [1] and (b) procedures with a maximum tolerated imbalance (MTI procedures) [23]. Within this paper we consider the following randomization procedures:

(I) Complete randomization (CR): Patients are randomized to treatments by tossing a fair coin.
(I) Efron’s biased coin design with bias probability *p* (EBC(*p*)) [24]: Patients are randomized to treatments by coin toss. The coin is biased with probability *p* in favor of the less frequently assigned treatment or fair whenever the same number of patients have been assigned to both treatments.
(IIab) Random allocation rule (RAR): Exactly *n*/2 patients are randomized to the control treatment and *n*/2 patients to the experimental treatment so that final balance is achieved with a maximum tolerated imbalance of *n*/2. All possible randomization sequences are equally likely.
(IIab) Permuted block randomization with block size *k* (PBR(*k*)): Patients are randomized in blocks of size *k*, with a random allocation rule being used within each block. The procedure achieves final balance and has a maximum tolerated imbalance of *k*/2.
(IIab) Berger’s maximal procedure with final balance and a maximum tolerated imbalance of *b* (MP(*b*)) [25]: The set of randomization sequences generated by the random allocation rule is restricted to those for which the imbalance in group sizes never exceeds *b*. Among those sequences, all are equally likely.
(IIb) Big stick design with a maximum tolerated imbalance of *b* (BSD(*b*)) [26]: Patients are randomized to treatments by tossing a fair coin. If the imbalance in group sizes reaches a maximum tolerated imbalance *b*, the next patient is deterministically assigned to the treatment with fewer patients.
(IIb) Chen’s biased coin design with a maximum tolerated imbalance of *b* and bias probability of *p* (CHEN(*b*, *p*)) [27]: Patients are randomized to treatments using Efron’s biased coin design with bias probability *p*. If the imbalance in group sizes reaches *b*, the next patient is deterministically assigned to the treatment with fewer patients.

Let *h*_*C*_ and *h*_*E*_ denote the hazard functions associated with the control and experimental treatment. Assuming independent survival times and a constant hazard ratio over time, i.e., *HR* = *h*_*E*_(*t*)/*h*_*C*_(*t*) for all *t* > 0, the log-rank test [21, 22] can be used to test the hypothesis that *HR* = 1. Conditioning on the number of patients at risk within the control and experimental group before each event, let *O*_*j*_ denote the number of observed events and *e*_*j*_ the number of expected events in the control group at the *j*th event time *τ*_*j*_ for *j* ∈ {1, …, *d*}. Under the null hypothesis, the log-rank statistic asymptotically follows a standard normal distribution [28], that is

$$\begin{array}{c} LR\left(z\right)=\frac{\sum_{j=1}^{d}\left(O_{j}-e_{j}\right)}{\sqrt{\sum_{j=1}^{d}e_{j}\left(1-e_{j}\right)}}\;\overset{asymp.}{∼}\;N\left(0,1\right). \end{array}$$ (1)

Under the alternative hypothesis, the log-rank statistic asymptotically follows a non-standard normal distribution. Possible approaches to obtain approximations for the expected value and variance have for example been proposed by Schoenfeld (1981) [29] or Freedman (1982) [30].

## Bias model

The following bias model is a generalization of the model described in [9] which also incorporates time trends and can be applied not only to exponentially distributed survival times. For a randomization sequence ***z***, let *h_i_*(*t*, ***z***) denote the hazard function of patient *i* at time *t*. We assume that the hazard function is affected by a biased selection of patients and an unobserved time trend such that

$$\begin{array}{c} h_{i}\left(t,z\right)=h_{C}\left(t\right)\;\text{exp}\;(z_{i}\;\text{ln}\left(HR\right)+\eta_{i}\left(z\right)+\theta_{i}), \end{array}$$ (2)

where *η_i_*(***z***) and *θ*_*i*_ are functions that quantify the strength of selection and chronological bias. In contrast to the additive bias model for continuous outcomes [10], this newly defined bias model for survival outcomes has a multiplicative effect on the hazard functions.

### Selection bias

We consider the phenomenon of allocation bias [31], a specific type of selection bias, using the model assumptions from [14, 15]: We assume that all previously randomized allocations are unblinded and therefore known to the investigator, as is the intended allocation ratio of 1:1. Since knowledge of previous allocations may enable an investigator to predict upcoming allocations, this bears the risk for a biased selection of patients. Further, we assume that the investigator guesses upcoming treatments independently of the underlying randomization procedure, employing the convergence strategy by Blackwell and Hodges [14]. Let η∈R be a factor for the strength of selection bias. Based on the observed number of allocations to the control $N_{i-1}^{C}\left(z\right)$ and experimental $N_{i-1}^{E}\left(z\right)$ treatment before the enrollment of patient *i*, we assume that the investigator predicts the next treatment whenever there is an imbalance in group sizes. This translates into the following biasing strategy

$$\begin{array}{c} \eta_{i}\left(z\right)=\eta \mathrm{sgn}(N_{i-1}^{E}\left(z\right)-N_{i-1}^{C}\left(z\right)), \end{array}$$

where sgn is the sign function. Note that for *η* = 0, there is no selection bias effect on the hazard rate from Eq (2). Assume that long survival is desirable. Then, for any randomization procedure targeting balanced group sizes, an effect of *η* > 0 reflects the situation where the hazard rate of control patients is worsened in expectation while the hazard rate of experimental patients is improved in expectation. Conversely, an effect of *η* < 0 equates a biasing strategy where the hazard rate of control patients is improved in expectation while the hazard rates of experimental patients is worsened in expectation.

The right choice of a selection bias function depends primarily on whether the blinding of the investigator can be guaranteed. If it can be excluded with certainty that the investigator will become aware of past treatment assignments, for example if enrollment and randomization are carried out by an external institution, it is permissible to assume no selection bias at all. However, this very strict assumption will not be fulfilled in most studies. If blinding cannot be implemented or is at risk, a selection bias function should be defined. The selection bias effect *η* characterizes the heterogeneity of the medical condition within the study population and should be chosen accordingly. An assessment of the heterogeneity can be made on the basis of clinical experience or the anticipated treatment effect and by taking into account the precision of the inclusion and exclusion criteria.

### Chronological bias

There are multiple causes for chronological bias in the form of time trends, such as improved performance of the treating physician or staff (learning curve), or an amendment of the enrollment criteria [7, 32]. Following [4], possible options for the time trend function *θ*_*i*_ include stepwise, linear, or logarithmic time trends

$$\begin{array}{ll} \text{Stepwise} & \theta_{i}=\theta \;1_{\left\{i>k\right\}},\;k\in \left\{1,\ldots ,n-1\right\},\\ \text{Linear} & \theta_{i}=\theta \;(i-1)/(n-1),\\ \text{Logarithmic} & \theta_{i}=\theta \;\text{ln}(i)/\;\text{ln}(n), \end{array}$$

where θ∈R is a parameter that quantifies the strength and direction of the underlying time trend. Assuming long survival is desirable, an effect of *θ* > 0 indicates that the hazard rate worsens over the course of the trial, while, conversely, *θ* < 0 indicates that the hazard rate improves over the course of the trial. For *θ* = 0 no time trend effect is assumed.

The choice of a time trend function should be based on the anticipated temporal changes that could affect the treatment success. For example, the assessment of whether a learning curve can occur depends on the experience of the treating physicians as well as on the novelty of the method under investigation. The risk assessment for an amendment of inclusion criteria or a change in classification of a disease due to medical progress should be related to the length of the accrual period. After a time trend function has been selected, a suitable time trend effect *θ* must be specified. If data from similar studies are available, the time trend effect can be estimated from these observations. If no such data is available, the time trend effect can be quantified on the basis of medical experience or the anticipated treatment effect.

## Influence of bias on the log-rank statistic

If the survival distributions of the patients are influenced by selection or chronological bias as introduced in our bias model from Eq (2), this also affects the distribution of the log-rank statistic. We derive an approximation formula to compute the rejection probabilities in the presence of bias depending on the randomization sequence. The dependence of the type I error probability on the sample size, the bias effects and the randomization procedure is showcased in a sensitivity analysis. We conclude with a case study to illustrate how our evaluation method can be applied to select a suitable randomization procedure on the basis of the expected type I error probability. All computations were performed on a computer with an Intel i7-4710MQ CPU Quad-core (2.5 GHz) and 16 GB RAM under a Windows 7 (64-bit) operating system. The code was written in R version 3.5.1. [33], using the randomizeR package version 2.0 [34].

### Distribution of the log-rank statistic in the presence of bias

Conditioning on the realized randomization sequence ***z***, we demonstrate that if ln(*HR*) is in $O(n^{-1/2})$ and (*θ_i_* + *η_i_*(***z***)) can be expressed in terms of ln(*HR*), the log-rank statistic is asymptotically normal with the mean depending on ***z*** and variance 1, i.e.,

$$\begin{array}{c} LR\left(z\right)\;\;\overset{asymp.}{∼}\;N\left(E_{bias}\left(z\right),1\right). \end{array}$$ (3)

The arguments used are the same as those used by Schoenfeld [29]. Let *S*_*i*_ and *f*_*i*_ denote the survival distribution and density function corresponding to the *i*th patient where *i* ∈ {1, …, *n*}. The validity of Eq (3) can be shown by conditioning on the realized randomization sequence ***z*** = (*z*_1_, …, *z_n_*), as well as the information as to which patients are at risk before event time *τ*_*j*_, *j* = 1, …, *d*. Assume that ln(*HR*) is in $O(n^{-1/2})$ and that the sum of the two bias functions (*θ_i_* + *η_i_*(***z***)) can be expressed in terms of ln(*HR*). As in Eq (1), let *e_j_*(***z***) denote the expected number of events at event time *τ*_*j*_ in the control group under the null hypothesis and let *μ_j_*(***z***) be the true expected number of events under the bias model (2). Then, if *Y*_1_, …, *Y*_*n*_ are the observed event or censoring times

$$\begin{array}{c} e_{j}\left(z\right)=\frac{\sum_{i=1}^{n}1_{\left\{Y_{i}\geq \tau_{j}\right\}}\left(1-z_{i}\right)}{\sum_{i=1}^{n}1_{\left\{Y_{i}\geq \tau_{j}\right\}}},\;\mu_{j}\left(z\right)=\frac{\sum_{i=1}^{n}h_{i}\left(\tau_{j},z\right)1_{\left\{Y_{i}\geq \tau_{j}\right\}}\left(1-z_{i}\right)}{\sum_{i=1}^{n}h_{i}\left(\tau_{j},z\right)1_{\left\{Y_{i}\geq \tau_{j}\right\}}}. \end{array}$$

By rewriting *μ_j_*(***z***) as a function of ln(*HR*) and expanding in a Taylor series around zero, it can be shown the log-rank statistic convergences in probability to

$$\begin{array}{c} \frac{\sum_{j=1}^{d}\left(O_{j}-\mu_{j}\left(z\right)\right)}{\sqrt{\sum_{j=1}^{d}\mu_{j}\left(z\right)\left(1-\mu_{j}\left(z\right)\right)}}+\frac{\sum_{j=1}^{d}\left(\mu_{j}\left(z\right)-e_{j}\left(z\right)\right)}{\sqrt{\sum_{j=1}^{d}e_{j}\left(z\right)\left(1-e_{j}\left(z\right)\right)}}. \end{array}$$ (4)

The first term from (4) is asymptotically standard normal, thus yielding the asymptotic variance of 1. To obtain the asymptotic value of the second term consider the two functions

$$\begin{array}{c} \pi \left(t,z\right)=\frac{\sum_{i=1}^{n}\left(1-z_{i}\right)S_{i}\left(t,z\right)}{\sum_{i=1}^{n}S_{i}\left(t,z\right)},\;\varphi \left(t,z\right)=\frac{\sum_{i=1}^{n}\left(1-z_{i}\right)f_{i}\left(t,z\right)}{\sum_{i=1}^{n}f_{i}\left(t,z\right)}. \end{array}$$

Then *e_j_*(***z***) can be approximated as

$$\begin{array}{c} e_{j}\left(z\right)\approx \frac{\sum_{i=1}^{n}S_{i}\left(\tau_{j},z\right)S_{cen}\left(\tau_{j}\right)\left(1-z_{i}\right)}{\sum_{i=1}^{n}S_{i}\left(\tau_{j},z\right)S_{cen}\left(\tau_{j}\right)}=\pi \left(\tau_{j},z\right), \end{array}$$

and *μ_j_*(***z***) as

$$\begin{array}{c} \mu_{j}\left(z\right)\approx \frac{\sum_{i=1}^{n}h_{i}\left(\tau_{j},z\right)S_{i}\left(\tau_{j},z\right)S_{cen}\left(\tau_{j}\right)\left(1-z_{i}\right)}{\sum_{i=1}^{n}h_{i}\left(\tau_{j},z\right)S_{i}\left(\tau_{j},z\right)S_{cen}\left(\tau_{j}\right)}=\frac{\sum_{i=1}^{n}f_{i}\left(\tau_{j},z\right)\left(1-z_{i}\right)}{\sum_{i=1}^{n}f_{i}\left(\tau_{j},z\right)}=\varphi \left(\tau_{j},z\right). \end{array}$$

Expanding the second term from (4) by 1/*n* and applying the law of large numbers yields:

$$\begin{array}{cc} E_{bias}\left(z\right) & \approx \frac{1/n\sum_{j=1}^{d}\left(\varphi \left(\tau_{j},z\right)-\pi \left(\tau_{j},z\right)\right)}{\sqrt{1/n\sum_{j=1}^{d}\pi \left(\tau_{j},z\right)\left(1-\pi \left(\tau_{j},z\right)\right)}}\sqrt{n}\\ & \approx \frac{\int_{0}^{\infty }\left(\varphi \left(t,z\right)-\pi \left(t,z\right)\right)V\left(t,z\right)\;dt}{\sqrt{\int_{0}^{\infty }\pi \left(t,z\right)\left(1-\pi \left(t,z\right)\right)V\left(t,z\right)\;dt}}\sqrt{n}, \end{array}$$

where *V*(*t*, ***z***) is the mixture density

$$\begin{array}{c} V\left(t,z\right)=\frac{\sum_{i=1}^{n}f_{i}\left(t,z\right)S_{cen}\left(t\right)S_{uni}\left(t\right)}{n}, \end{array}$$

with *S*_*cen*_ denoting the survival function of the random censoring mechanism and *S*_*uni*_ the survival function of the censoring mechanism due to end of follow-up, i.e.,

$$\begin{array}{c} S_{uni}\left(t\right)=\{\begin{array}{cc} 1,\; & \text{for}\;t<(F-A),\\ 1-(t-(F-A))/A,\; & \text{for}\;(F-A)\leq t<F,\\ 0,\; & \text{for}\;t\geq F. \end{array} \end{array}$$

Consequently, if selection or chronological bias are present and the equality of two survival distributions is investigated using the log-rank test, formula (3) can be used to obtain an approximation of the rejection probability depending on the randomization sequence ***z***.

### Sensitivity analysis

The influence of the randomization procedure on the rejection probability of the log-rank test in the presence of selection or chronological bias is shown for different bias effects and sample sizes to illustrate the dependence on the study setting. We limit our considerations to scenarios in which the null hypothesis is true, i.e., the rejection probability corresponds to the type I error probability. The scenarios are simulated by drawing a Monte-Carlo sample of randomization sequences for each randomization procedure. The scenario dependent type I error probability of each randomization sequence is then calculated using approximation (3). The number of randomization sequences per Monte-Carlo sample is chosen to ensure a precision of at least 2 ⋅ 10^−4^ for the expected type I error probability with 99.5% certainty.

Two randomization procedures, PBR(8) and RAR, are compared in four scenarios with different sample sizes and bias settings. The comparison includes a small sample size of *n* = 40 and a large sample size of *n* = 200. We consider two different bias settings, one with a larger time trend and one with a stronger selection bias effect:

- **CB-Setting**: A linear time trend of *θ* = −0.6 and a selection bias effect of *η* = 0.05.
- **SB-Setting**: A stepwise time trend of *θ* = −0.05 after *k* = 0.5*n* and a selection bias effect of *η* = 0.1.

The common assumptions regarding the study design on which all four scenarios are based are shown in Table 1.

**Table 1:** Common assumptions of all four scenarios considered in the sensitivity analysis.

Parameter	Notation	Value
Significance level	*α*	5%
Length of accrual period	*A*	30 weeks
Total duration of the study	*F*	90 weeks
Common exponential dropout rate	λ_*cen*_	0.02
Exponential hazard rates	λ_*C*_, λ_*E*_	0.06

**Fig 1:**
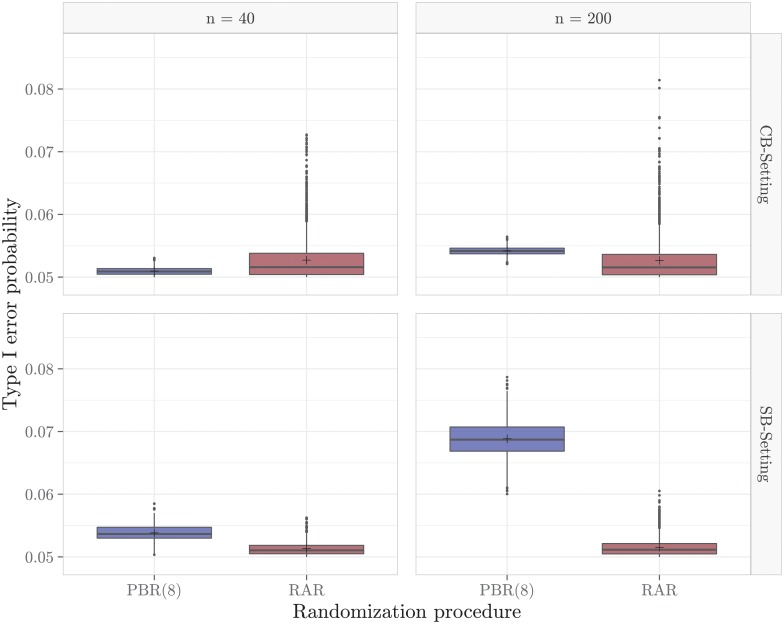
Influence of the study setting on the distribution of type I error probabilities. The distributions are based on a sample of 2,500 randomization sequences per randomization procedure.

The distributions of type I error probabilities of PBR(8) and RAR for the different scenarios are shown in Fig 1. On the one hand, there are clear differences between the different randomization sequences within a randomization procedure, as can be seen from the variation in type I error probabilities. On the other hand, there are also considerable differences between the two randomization procedures and between the different scenarios under consideration. The differences between PBR(8) and RAR are caused by the dependence of the rejection probability on the randomization sequence. This is because the set of randomly drawn randomization sequences depends on the probability distribution of the randomization procedure used. The differences between the SB- and CB-setting confirm that selection and chronological bias act differently on the type I error probability of the log-rank test. With regard to the sample size, the results of RAR seem to be similar while the rejection probability for *n* = 200 compared to *n* = 40 increases greatly in the case of PBR(8). Consequently, the rejection probabilities can also be affected by the sample size.

In summary, we observe that the presence of selection and chronological bias affects the type I error probability of the log-rank test. The extent of this influence depends on the type of bias, the sample size and on the randomization sequence. Due to the dependence on the randomization sequence, there is a dependence on the randomization procedure used. For this reason, the choice of the randomization procedure has a direct effect on the expected type I error probability of the log-rank test and thus on the susceptibility to selection and chronological bias. The results of the four scenarios show that, depending on the interaction of bias and sample size, a different randomization procedure should be chosen with respect to the expected type I error probability. Consequently, no general recommendation for a randomization procedure can be made and a suitable randomization procedure must always be chosen individually with regard to the study in question.

### Case study for acute myelogenous leukemia

We illustrate the application of our bias model in a case study which is based on the *aml* data set from the boot package [35] in R version 3.5.1. [33]. The choice of the case study is motivated by our desire to allow researchers to reproduce our results. The metric considered for choosing a suitable randomization procedure is the expected type I error probability [15]. Our simulation is designed to ensure a precision of at least 2 ⋅ 10^−4^ for the expected type I error probability with 99.5% certainty. This requirement results in 7,500 randomization sequences per randomization procedure. The rejection probability for each sequence is computed using approximation (3).

For comparison, we include variations of the aforementioned seven randomization procedures. Specifically, we consider CR, EBC(2/3), PBR(4), PBR(8), PBR(16), RAR, MP(3), MP(7), MP(11), BSD(3), BSD(7), BSD(11), CHEN(3, 2/3), CHEN(7, 2/3), and CHEN(11, 2/3), where the parameters are selected for the following reasons: According to Efron’s suggestion, the bias probability of Efron’s biased coin design and Chen’s biased coin design is defined as 2/3 [24]. The block size of 4 is chosen as an example of permuted block randomization with small block size, the block size of 8 as one with medium block size, and the block size of 16 as one with large block size. Finally, the maximum tolerated imbalance of all MTI procedures is set to 3, 7 and 11, so that the maximum loss of power due to imbalance is at most 0.5%, 1%, and 2%, respectively (determined using nQuery Advisor version 7.0).

#### Setting

We consider a fictitious study that aims at assessing the efficacy of maintenance chemotherapy consisting of cytarabine and 6-thioguanine for acute myelogenous leukemia. The effect estimates are based on the differences observed in a study by Embury *et al*. [35, 36]. The study population consists of patients with acute myelogenous leukemia (AML) who have never been treated for AML and who are in a state of complete remission after induction therapy. The primary outcome is the time a patient remains in complete remission (in weeks) and a difference between maintenance chemotherapy and control treatment (no maintenance chemotherapy) shall be detected with a power of 80% at a significance level of *α* = 5%. Using the *aml* data set, the estimated hazard ratio is *HR* = 0.4003. Under the assumption of exponentially distributed survival times, the hazard rate for the control treatment is estimated as λ_*C*_ = 0.0431, which results in a hazard rate of λ_*E*_ = 0.0173 for maintenance chemotherapy. The study is designed for an accrual period of 18 weeks and a total duration of 52 weeks. The common exponential dropout rate throughout the study is assumed to be λ_*cen*_ = 0.0077, thus, resulting in a total required sample size of *n* = 64 patients (determined using nQuery Advisor version 7.0). A summary of the study design is shown in Table 2.

**Table 2:** Setting of the AML study.

Parameter	Notation	Value
Significance level	*α*	5%
Nominal power	1 − *β*	80%
Sample size	*n*	64
Length of accrual period	*A*	18 weeks
Total duration of the study	*F*	52 weeks
Common exponential dropout rate	λ_*cen*_	0.0077
Hazard rate maintenance chemotherapy	λ_*E*_	0.0173
Hazard rate control treatment	λ_*C*_	0.0431
Hazard ratio	*HR*	0.4003
Selection bias effect	*η*	0.1831
Logarithmic time trend effect	*θ*	-0.1144

#### Considerations relating to the randomization procedure

To achieve a state of complete remission, all patients receive induction therapy before continuing with either maintenance chemotherapy or no further chemotherapy. It is suspected that, in addition to the maintenance chemotherapy, the induction therapy also has an influence on the time a patient remains in complete remission [36]. Furthermore, it was observed that the duration of induction therapy until complete remission could be significantly shortened by changes in treatment regime [36]. This indicates that there is a risk of a learning curve or other medical progress in induction therapy. To account for this uncertainty, we define a small logarithmic time trend effect:

$$\begin{array}{c} \theta_{i}=0.125\;\text{ln}\left(HR\right)\;\text{ln}\left(i\right)/\;\text{ln}\left(n\right). \end{array}$$

This time trend function reflects the situation where the maximum difference in treatment success, attained between the first and last patient enrolled, is of magnitude one eighth as strong as the treatment effect (*θ* = 0.125 ln(*HR*)).

With regard to the definition of a selection bias function, the following considerations should be taken into account: The concealment of past treatment assignments is not possible, as it is impossible to conceal whether or not a patient received chemotherapy and a placebo chemotherapy would be unethical. Since the only inclusion criterion for patients is to be in a state of complete remission after induction therapy for acute myelogenous leukemia, the study population will be very heterogeneous. For this reason, a strong selection bias effect, one fifth as strong as the anticipated treatment effect is assumed (*η* = 0.2 ln(*HR*)):

$$\begin{array}{c} \eta_{i}\left(z\right)=0.2\;\text{ln}\left(HR\right)\;\text{sgn}(N_{i-1}^{E}\left(z\right)-N_{i-1}^{C}\left(z\right)). \end{array}$$

#### Evaluation of the randomization procedures

The mean type I error probabilities and standard deviations corresponding to each randomization procedure are summarized in Table 3. In general, the mean type I error probability is increased compared to the nominal significance level of 5%. The randomization procedures which display the smallest increase are CR, BSD(7), and BSD(11) with mean type I error probabilities of 5.2%. RAR, MP(11), and MP(7) perform only slightly inferior with mean type I error probabilities of 5.4%, 5.4%, and 5.5%, respectively. The increase is most severe for PBR(4) with a mean type I error probability of 8.1%. Based on the expected type I error probabilities, one of the randomization procedures CR, BSD(7), or BSD(11) is most suitable for the given survival study. Since the maximum loss of power due to imbalance is best controlled by BSD(7), this gives BSD(7) an advantage over CR and BSD(11).

**Table 3:** Summary statistics of type I error probabilities by randomization procedure.

Randomization procedure	mean value	standard deviation
BSD(3)	0.055	0.003
BSD(7)	0.052	0.003
BSD(11)	0.052	0.002
CHEN(3, 2/3)	0.065	0.006
CHEN(7, 2/3)	0.062	0.006
CHEN(11, 2/3)	0.062	0.007
CR	0.052	0.002
EBC(2/3)	0.062	0.006
MP(3)	0.062	0.005
MP(7)	0.055	0.004
MP(11)	0.054	0.004
PBR(4)	0.081	0.004
PBR(8)	0.070	0.005
PBR(16)	0.062	0.005
RAR	0.054	0.004

The summary statistics are based on a sample of 7,500 randomization sequences per randomization procedure.

## Discussion

Although it is widely acknowledged by regulatory authorities that well-conducted randomized controlled trials yield a higher level of evidence compared to observational studies [37–39], the importance attributed to randomization itself is usually limited to good implementation rather than a well conceived design [10]. This is reflected in the predominant use of permuted block randomization [40], which is usually chosen without a sound explanation [7, 23]. For this reason, we strive to strengthen awareness of the fact that the choice of randomization procedure affects the extent to which a study is susceptible to certain types of bias. This was confirmed both in our sensitivity analysis and in the considered case study. In order to enable scientists to select a randomization procedure that is suitable for their individual study, appropriate bias models and easy-to-use evaluation methods are needed.

Our results extend previous evaluation methods for normally and exponentially distributed outcomes to the simultaneous consideration of selection and chronological bias in survival studies with an accrual and follow-up period as well as different types of censoring. In doing so, the consideration of selection and chronological bias is not necessarily limited to the bias model considered here. For example, it is possible to define different time trend functions to reflect other more complex study settings. Likewise, one can consider alternative biasing strategies, e.g., under the assumption that the investigator guesses upcoming allocations taking into account the conditional allocation probabilities of the employed randomization procedure [8, 12]. The presented evaluation method addresses the influence on the distribution of the log-rank statistic, since the impact of bias on the test decision is one of the most important measures [7]. While the metric we used was the expected type I error probability, other evaluation metrics such as the power can also be considered for quantifying the performance of a randomization procedure [10].

The most commonly used methods for analyzing survival data are the Cox proportional hazards model and the log-rank test [19, 20], which in turn is equivalent to the score test of the discrete Cox proportional hazards model when the treatment group is the only covariate considered [41]. The approximation formula can thus also be used to gain an impression of the impact of bias if the final analysis shall be carried out by using a Cox regression model. Therefore, the proposed evaluation method is applicable to a majority of survival studies. We have shown that the assessment of whether and to what extent selection and chronological bias pose a risk must be carried out individually for each survival study at the trial planning stage. For this reason, we recommend to always compare different randomization procedures before conducting a clinical study. This leads to an improved study design and thus serves the greater goal of increasing the level of evidence. By implementing our results in the R-package randomizeR [34], we provide a free software tool that makes such a comparison possible for everyone.

## Conclusion

The presented results enable researchers in the planning phase of a survival study to make a scientifically sound choice of a randomization design. Due to the frequent use of the log-rank test and Cox’s proportional hazards model, our approach is applicable in most scenarios.

## Supporting information

S1 File R-code of the sensitivity analysis. This code can be used to perform the sensitivity analysis and generate the box plots from Fig 1.
(R) Click here for additional data file.

S2 File R-code of the case study. This code can be used to generate the results from the case study.
(R) Click here for additional data file.

S3 File R source package randomizeR_2.0.tar. Source code of the randomizeR package version 2.0.
(GZ) Click here for additional data file.

## References

[1] Rosenberger WF, Lachin JM. Randomization in clinical trials: Theory and Practice Wiley Series in Probability and Statistics. Hoboken, New Jersey: John Wiley & Sons; 2015.

[2] Senn SS. Seven myths of randomisation in clinical trials. Statistics in Medicine. 2013;32:1439–1450. 10.1002/sim.5713 23255195

[3] Matts JP, McHugh RB. Analysis of accrual randomized clinical trials with balanced groups in strata. Journal of chronic disease. 1978;31:725–740. 10.1016/0021-9681(78)90057-7 748369

[4] Tamm M, Hilgers RD. Chronological bias in randomized clinical trials arising from different types of unobserved time trends. Methods of Information in Medicine. 2014;53:501–510. 10.3414/ME14-01-0048 25396221

[5] Devereaux PJ, Bhandari M, Clarke M, Montori VM, Cook DJ, Yusuf S, et al Need for expertise based randomised controlled trials. BMJ. 2005;330:88 10.1136/bmj.330.7482.88 15637373 PMC543877

[6] Hopper AN, Jamison MH, Lewis WG. Learning curves in surgical practice. Postgraduate Medical Journal. 2007;83:777–779. 10.1136/pgmj.2007.057190 18057179 PMC2750931

[7] ICH Topic E9. Statistical principles for clinical trials; 1998; Accessed: 2018-05-03. Available from: http://www.ema.europa.eu/docs/en_GB/document_library/Scientific_guideline/2009/09/WC500002928.pdf.

[8] Berger VW. Selection bias and covariate imbalances in randomized clinical trials Statistics in Practice. Chichester, England: John Wiley & Sons; 2005.

[9] Rückbeil MV, Hilgers RD, Heussen N. Assessing the impact of selection bias on test decisions in trials with a time-to-event outcome. Statistics in Medicine. 2017;36:2656–2668. 10.1002/sim.7299 28417471 PMC5516162

[10] Hilgers RD, Uschner D, Rosenberger WF, Heussen N. ERDO—A framework to select an appropriate randomization procedure for clinical trials. BMC Medical Research Methodology. 2017;17:251–257. 10.1186/s12874-017-0428-z PMC5715815 29202708

[11] Kennes LN, Cramer E, Hilgers RD, Heussen N. The impact of selection bias on test decisions in randomized clinical trials. Statistics in Medicine. 2011;30:2573–2581. 10.1002/sim.4279 21717489

[12] Tamm M, Cramer E, Kennes LN, Heussen N. Influence of selection bias on the test decision. A simulation study. Methods of Information in Medicine. 2012;51:138–143. 10.3414/ME11-01-0043 22101391

[13] Langer S. The modified distribution of the t-test statistic under the influence of selection bias based on random allocation rule. RWTH Aachen University; 2014.

[14] Blackwell D, Hodges JL. Design for the control of selection bias. The Annals of Mathematical Statistics. 1957;28:449–460. 10.1214/aoms/1177706973

[15] Proschan M. Influence of selection bias on type I error rate under random permuted block designs. Statistica Sinica. 1994;4:219–231.

[16] Berger VW. Quantifying the magnitude of baseline covariate imbalances resulting from selection bias in randomized clinical trials. Biometrical Journal. 2005;47:119–127. 10.1002/bimj.200410106 16389910

[17] Uschner D, Hilgers RD, Heussen N. The Impact of Selection Bias in Randomized Multi-Arm Parallel Group Clinical Trials. Plos One. 2018;13 10.1371/journal.pone.0192065 29385190 PMC5792025

[18] Bland JM, Altman DG. The logrank test. British Medical Journal. 2004;328:1073 10.1136/bmj.328.7447.1073 15117797 PMC403858

[19] Clark TG, Bradburn MJ, Love SB, Altman DG. Survival Analysis Part I: Basic concepts and first analyses. British Journal of Cancer. 2003;89:232–238. 10.1038/sj.bjc.6601118 12865907 PMC2394262

[20] Bradburn MJ, Clark TG, Love SB, Altman DG. Survival Analysis Part II: Multivariate data analysis—an introduction to concepts and methods. British Journal of Cancer. 2003;89:431–436. 10.1038/sj.bjc.6601119 12888808 PMC2394368

[21] Mantel N. Evaluation of survival data and two new rank order statistics arising in its consideration. Cancer Chemotherapy Reports. 1966;50:163–170. 5910392

[22] Peto R, Peto J. Asymptotically efficient rank invariant test procedures. Journal of the Royal Statistical Society, Series A. 1972;135:185–207. 10.2307/2344317

[23] Berger VW, Bejleri K, Agnor R. Comparing MTI randomization procedures to blocked randomization. Statistics in Medicine. 2016;35:685–694. 10.1002/sim.6637 26337607

[24] Efron B. Forcing a Sequential Experiment to be Balanced. Biometrika. 1971;58:403–417. 10.1093/biomet/58.3.403

[25] Berger VW, Ivanova A, Knoll MD. Minimizing predictability while retaining balance through the use of less restrictive randomization procedures. Statistics in Medicine. 2003;22:3017–3028. 10.1002/sim.1538 12973784

[26] Soares JF, Wu CFJ. Some Restricted randomization rules in sequential designs. Communications in Statistics—Theory and Methods. 1983;12:2017–2034. 10.1080/03610928308828586

[27] Chen YP. Biased coin design with imbalance tolerance. Communications in Statistics Stochastic Models. 1999;15(5):953–975. 10.1080/15326349908807570

[28] Kalbfleisch JD, Prentice RL. The statistical analysis of failure time data Wiley series in probability and mathematical statistics: Applied probability and statistics. New York, USA: John Wiley & Sons; 1980.

[29] Schoenfeld DA. The asymptotic properties of nonparametric tests for comparing survival distributions. Biometrika. 1981;68:316–319. 10.1093/biomet/68.1.316

[30] Freedman LS. Tables of the number of patients required in clinical trials using the logrank test. Statistics in Medicine. 1982;1:121–129. 10.1002/sim.4780010204 7187087

[31] Nunan D, Heneghan C, Spencer EA. Catalogue of bias: allocation bias. BMJ Evidence-Based Medicine. 2018;23:20–21. 10.1136/ebmed-2017-110882 29367320

[32] Catalogue of Bias Collaboration, Spencer EA, Heneghan C. Chronological bias. In: Catalogue Of Bias 2017; Accessed: 2018-12-20. Available from: https://catalogofbias.org/biases/chronological-bias/.

[33] R Core Team. R: A Language and Environment for Statistical Computing; 2013 Available from: http://www.R-project.org/.

[34] Schindler D, Uschner D, Manolov M, Pham TM, Hilgers RD, Heussen N. randomizeR: Randomization for Clinical Trials; 2016.

[35] Canty A, Ripley BD. boot: Bootstrap R (S-Plus) Functions; 2017. Available from: https://CRAN.R-project.org/package=boot.

[36] Embury SH, Elias L, Hood PH, Greenberg CE, Schrier SL. Remission maintenance therapy in acute myelogenous leukaemia. Western Journal of Medicine. 1977;126:267–272. 266313 PMC1237541

[37] Canadian Task Force on the periodic Health Examination. The periodic health examination. Canadian Medical Association. 1979;121:1193–1254. PMC1704686 115569

[38] Burns PB, Rohrich RJ, Chung KC. The Levels of Evidence and their role in Evidence-Based Medicine. Plastic and Reconstructive Surgery. 2011;128:305–310. 10.1097/PRS.0b013e318219c171 21701348 PMC3124652

[39] ICH Topic E8. General Considerations for Clinical Trials; 1998; Accessed: 2018-05-03. Available from: http://www.ema.europa.eu/docs/en_GB/document_library/Scientific_guideline/2009/09/WC500002877.pdf.

[40] Senn SS. Statistical Issues in Drug Development Statistics in Practice. Hoboken, New Jersey: John Wiley & Sons; 2008.

[41] Collett D. Modelling Survival Data in Medical Research Chapman & Hall/CRC Texts in Statistical Science. Chichester, England: Taylor & Francis; 2003.

