# Supplementary material for: Randomization in survival studies: An evaluation method that takes into account selection and chronological bias
Source: PLoS One. 2019 Jun 3;14(6):e0217946. doi: 10.1371/journal.pone.0217946 (PMC6546249; doi:10.1371/journal.pone.0217946)
Supplement: S3 File — Source code of the randomizeR package version 2.0. (GZ) [file pone.0217946.s003.gz › randomizeR/inst/doc/article.pdf]

randomizeR: An R Package for the Assessment and Implementation of Randomization in Clinical Trials

Diane Uschner
RWTH Aachen University

David Schindler
RWTH Aachen University

Nicole Heussen
RWTH Aachen University

Ralf-Dieter Hilgers
RWTH Aachen University

Abstract

This introduction to the R package **randomizeR** is a slightly modified version of Uschner, Schindler, Heussen, and Hilgers (2016).

Randomization in clinical trials is the key design technique to ensure the comparability of treatment groups. Although there exist a large number of software products which assist the researcher to implement randomization, no tool which covers a wide range of procedures and allows the comparative evaluation of the procedures under practical restrictions has been proposed in the literature.

The R package **randomizeR** addresses this need. The paper includes a detailed description of the **randomizeR** package that serves as a tutorial for the generation of randomization sequences and the assessment of randomization procedures.

Keywords: restricted randomization procedure, selection bias, chronological bias, R.

1. Introduction

Randomization is a design technique to ensure the comparability of treatment groups in clinical trials by introducing a deliberate element of chance. Armitage (1982) states the three main goals that are supposed to be achieved by randomization: First, it tends to balance known and unknown covariates and, thus, to produce structural equality of the treatment groups. Second, by ensuring effective blinding of treatment allocations from investigators and patients, randomization helps to avoid bias caused by the selection of patients. Finally, randomization contributes to the internal validity of a trial that provides the basis for statistical inference. The importance of randomization for clinical trials was first noted in the 1940s by Sir A. Bradford Hill (see Chalmers 1999) who realized that successful blinding of treatment allocations was impossible without randomization. Since that time, regulators have advocated the use of randomization in their guidelines (see for example ICH E9 1998) and several different randomization procedures have been proposed in the literature. It has been noticed that different randomization procedures behave differently, e.g., concerning their susceptibility to bias and their potential to control power and type-I-error probability. An overview containing the latest developments can be found in Rosenberger and Lachin (2016).

Despite the importance of randomization in the context of clinical trials, the novelties of the

last decades have hardly found their way into clinical practice. Berger, Bejleri, and Agnor (2015) remark that clinicians stick with randomization procedures that are easily available, although their poor properties have been widely discussed and better alternatives have been proposed. Notably, the most suitable randomization procedure may depend on the clinical context. For example, when block randomization is used, Tamm and Hilgers (2014) advocate small block sizes to control chronological bias, while Kennes, Hilgers, and Heussen (2012) show that larger blocks are better to control selection bias.

The choice of an adequate randomization procedure is crucial to achieve favorable properties such as control of power and type-I-error rate, especially for small sample sizes where asymptotic assumptions do not hold. The R package **randomizeR** assists clinical trialists in making a scientifically sound choice based on these criteria. It combines the assessment of randomization procedures with the generation of randomization lists for clinical trials. Existing software tools such as the **blockrand** package by Snow (2013) implement only a very limited number of randomization procedures. The special class of response-adaptive randomization procedures is not included in **randomizeR**, but has currently been implemented for trials with time-to-event outcome in the MTLAB package **RARtool** by Ryeznik, Sverdlov, and Wong (2015). To our knowledge, no software package for the assessment of the practical properties of randomization procedures exists, particularly not for the assessment of their exact properties in small clinical trials.

This article gives a detailed description of **randomizeR**. It is organized as follows. Section 2 gives the background for assessing the properties of randomization procedures, such as susceptibility to bias. The randomization procedures are presented in Section 2.3. Section 3 shows how **randomizeR** implements the methods from the background chapter. Particularly, Section 3.2.2 provides a tutorial for the generation of randomization lists with **randomizeR**, and 3.3.1 illustrates the assessment of randomization procedures with respect to the different types of bias and other criteria.

2. Assessing the properties of randomization procedures

2.1. Notation and model

We consider the case of a two armed clinical trial with parallel group design. Let A and B be treatments that influence a continuous outcome Y. A *randomization procedure* $\mathcal{M}$ is a probability distribution on the space $\Omega = \{0,1\}^N$. A *randomization sequence* is an element $t \in \Omega$, where $t_i = 1$ if patient i is allocated to treatment A and $t_i = 0$ otherwise. Let $T = (T_1, \dots, T_N)$ denote a random variable with probability distribution $\mathcal{M}$ taking values in Ω. Throughout the paper, let the outcome y_i of patient $i = 1, \dots, N$ be the realization of a normally distributed random variable

$$Y_i \sim \mathcal{N}(\mu_A \cdot T_i + \mu_B \cdot (1 - T_i), \sigma^2) \tag{1}$$

with group expectations μ_A, μ_B and equal but unknown variance $\sigma^2 > 0$. The outcome y_i is called *response*. Higher values of the response are regarded as better. We test the null hypothesis that the expectation of the experimental treatment A does not differ from the expectation of the control treatment B against the two-sided alternative

$$H_0 : \mu_A = \mu_B \quad \text{vs.} \quad H_1 : \mu_A \neq \mu_B. \tag{2}$$

Trend	Shape
Linear	$\tau(i, \vartheta) = i \cdot \vartheta$
Logarithmic	$\tau(i, \vartheta) = \log(\frac{i}{N}) \cdot \vartheta$
Step	$\tau(i, \vartheta) = 1_{\{i \geq n_0\}} \cdot \vartheta, n_0 \in \{1, \ldots, N\}$

Table 1: The three types of time trend included in **randomizeR**.

When the response Y_i of patient i is influenced by an unobserved quantity b_i, we call b_i *bias* of the i-th patient. Bias includes selection bias or chronological bias, or both, as proposed in the sections 2.2.1 and 2.2.2. We investigate how the randomization procedures manage the misspecification of the model

$$Y_i \sim \mathcal{N}(\mu_A \cdot T_i + \mu_B \cdot (1 - T_i) + b_i, \sigma^2) \tag{3}$$

with b_i the fixed bias of patient i.

2.2. Criteria for the assessment

The potential of a randomization procedure to control the impact of bias on the study results along with other exemplary criteria for the choice of a randomization procedure are summarized in this section. The assessment of the impact of bias is important if the form of the bias is unknown or the bias is unobserved.

Susceptibility to chronological bias

Changes in study environment, e.g., increased diagnostic potential, may impact the response to treatment over time. Unobserved time trends lead to a bias of the estimator of the treatment effect, for which Matts and McHugh (1978) used the term *chronological bias*. Chronological bias is a special case of accidental bias as introduced by Efron (1971). Efron investigated the effects of covariates that have been (unintentionally) ignored in the model. Although the underlying trend could in theory be included in the model, the bias is often unobserved, or the exact form is unknown. Furthermore, especially in small population groups, it is challenging to use models with many explanatory variables. Rosenkranz (2011) measured the impact of chronological bias by the distortion of the type-I-error rate of the t test when the responses are influenced by a trend $\tau(i, \vartheta)$:

$$Y_i \sim \mathcal{N}(\mu_A \cdot T_i + \mu_B \cdot (1 - T_i) + \tau(i, \vartheta), \sigma^2). \tag{4}$$

Tamm and Hilgers (2014) proposed three shapes of trend that are summarized in Table 1. Here log denotes the natural logarithm and $1_{\{i \geq n_0\}}$ is the indicator function yielding the value one if $i \geq n_0$ and zero if $i < n_0$.

Susceptibility to selection bias

Sir A. Bradford Hill was the first to adopt randomization in clinical trials (see Chalmers 1999). His aim was to ensure effective blinding and to avoid bias due to the conscious or unconscious selection of patients to treatment groups, the so-called *selection bias*. We consider

two measures for selection bias proposed in the literature, the expected number of correct guesses and the influence of selection bias on the test decision.

The expected number of correct guesses was introduced by Blackwell and Hodges (1957). They assume that, based on past treatment assignments, the investigator consciously or unconsciously guesses the next treatment based on the past assignments. Suppose the investigator guesses that patient $i > 1$ will receive treatment $g(t, i) \in \{A, B\}$ based on the previous assignments $(t_1, \dots, t_{i-1})$. The correct guesses of a randomization sequence is the number of assignments the investigator guesses correctly:

$$CG(t) = |\{i \in \{1, \dots, N\} : g(t, i) = t_i\}|. \tag{5}$$

Two guessing strategies were investigated by Blackwell and Hodges (1957). Under the *convergence strategy (CS)*, the investigator assumes that the next patient is assigned to the group that has so far been assigned less. Under the *divergence strategy (DS)* the experimenter assumes that the next patient is assigned to the treatment that has so far been observed more often. At the beginning of the trial and when there is a tie, the investigator guesses the next allocation at random.

The expected number of correct guesses $E(CG(t))$ reflects the predictability of a sequence $t \in \Omega$. The overall predictability of a randomization procedure $\mathcal{M}$ is given by the average proportion of correct guesses:

$$avpCG_{\mathcal{M}} := \frac{1}{N} \cdot E_{\mathcal{M}}(E(CG(t))).$$

Proschan (1994) proposed to measure the influence of selection bias on the test decision when the responses are biased as a result of selecting the patients following the convergence strategy:

$$Y_i \sim \mathcal{N}(\mu_A \cdot T_i + \mu_B \cdot (1 - T_i) - \text{sign}(D_i) \cdot \eta, \sigma^2), \tag{6}$$

where $D_i := D_i(T) = \sum_{j=1}^{i} T_j - \sum_{j=1}^{i}(1 - T_j)$ denotes the *imbalance* of a randomization sequence, $\text{sign}(x)$ denotes the sign function, and η denotes the selection effect. Tamm, Cramer, Kennes, and Heussen (2012) demonstrated the impact of selection bias for different values of the selection effect η.

Balancing behavior

According to ICH E9 (1998), it is desirable for a randomization procedure to balance the group sizes throughout the trial as well as at the end of the trial, while avoiding predictability. Table 2 summarizes the measures for the imbalance that have been proposed in the literature (see for example Atkinson 2014). According to Lachin (1988) imbalance may cause decreased power of the statistical test in case of continuous endpoint and homoscedasticity.

2.3. Randomization procedures

Randomization procedures can be described in terms of a restricted or unrestricted random walk (see Proschan 1994). The restrictions imposed on the random walk lead to different randomization procedures. In this section, we give a short overview about the randomization procedures that are implemented in **randomizeR**. For a comprehensive overview we refer to Rosenberger and Lachin (2016).

Imbalance	Formula
Final	D_N
Absolute final	$\lvert D_N \rvert$
Maximal	$\max_{i=1,\ldots,N} \lvert D_i \rvert$
Loss	$\frac{D_N^2}{N}$

Table 2: Imbalance measures implemented in **randomizeR**.

```
Warning: package 'survival' was built under R version 3.5.3
```

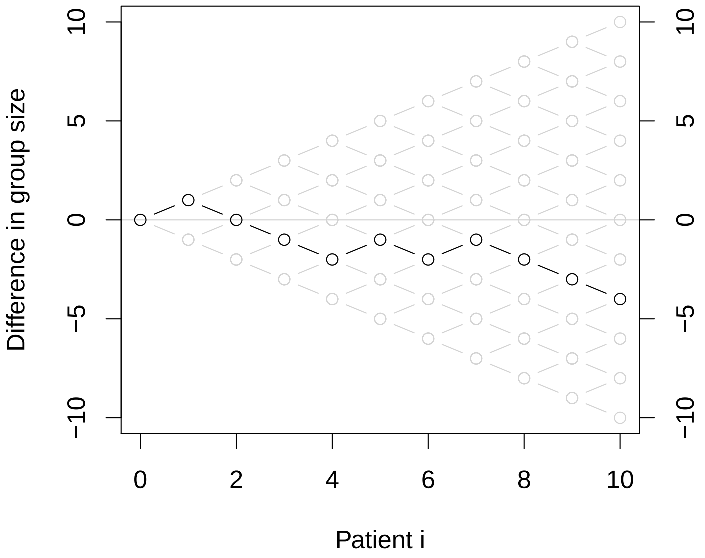

Figure 1: Random walk of the randomization sequences of CR.

Complete Randomization (CR) is equivalent to tossing a fair coin for the allocation of each patient. CR leads to 2^N equi-probable sequences where N denotes the total sample size. Figure 1 shows a randomization sequence produced by a CR in heavy black, along with all possible sequences in light gray.

Using Random Allocation Rule (RAR), patients are allocated by drawing N times without replacement from an urn consisting of $N/2$ balls for each treatment. RAR produces $\binom{N}{N/2}$ equi-probable sequences that all attain final balance.

Permuted Block Randomization (PBR) with *block constellation* $bc = (k_1, \dots, k_m)$ balances the allocations in the blocks of length $k_1, \dots, k_m$. For each block $j = 1, \dots, m$, an urn is filled with $k_j/2$ balls for each of the two treatments, and k_j balls are drawn without replacement from the urn. PBR leads to $\prod_{j=1}^{m} \binom{k_j}{k_j/2}$ equi-probable sequences that attain balance after each block, particularly at the end.

Permuted Block Randomization with *random block constellation* (RPBR) is similar to PBR, but the block constellation bc is sampled at random from the set of given block lengths rb. RPBR permits two variations (Heussen 2004; Rosenberger and Lachin 2016): The entries k_j of bc can either be drawn uniformly with replacement from rb until $\sum k_j \geq N$, or be conditioned to achieve final balance, namely $\sum k_j = N$.

The Truncated Binomial Design (TBD) consists of tossing a fair coin for the allocation of patients until $N/2$ heads or tails have occurred. The remaining patients are allocated deterministically to the opposite group. TBD results in sequences that attain final balance but are not equiprobable. TBD admits the same number of sequences as RAR. As an extension, TBD can be conducted in blocks similar to PBR, or similar to RPBR with random block constellation (RTBD).

The Maximal Procedure (MP) was proposed by Berger, Ivanova, and Knoll (2003). MP gives equal probability to all sequences that attain final balance and do not exceed a pre-specified maximum tolerated imbalance $mti \in \mathbb{N}$. Always when the imbalance boundary is reached, i.e., $|D_i| = mti$, a deterministic assignment is made to the underrepresented group in order to reduce the imbalance.

The Big Stick Design (BSD) introduced by Soares and Wu (1983) consists of tossing a fair coin until the imbalance $|D_i|$ reaches a maximum tolerated imbalance $mti \in \mathbb{N}$.

In Efron's Biased Coin design (EBC) (see Efron 1971), a biased coin is tossed for the next allocation in order to reduce the imbalance with probability $0.5 \leq p \leq 1$. When the groups are balanced, a fair coin is tossed.

Chen (1999) proposed a biased coin design with imbalance tolerance (CHEN) that combines BSD and EBC. When the groups are balanced, a fair coin is tossed for the allocation of patients. Otherwise, a biased coin with probability $0.5 \leq p \leq 1$ is tossed until reaching an imbalance boundary $mti \in \mathbb{N}$. When the imbalance boundary is reached, a deterministic assignment is made in order to reduce the imbalance.

The Accelerated Biased Coin Design (ABCD) proposed by Antognini and Giovagnoli (2004) uses the toss of a biased coin for the allocation of patients. The probability $p_i = p(D_{i-1}, a)$ for allocating the ith patient to the experimental group depends on the imbalance D_{i-1} and an *acceleration parameter* $a > 0$. The acceleration parameter exponentially weights the imbalance and thus determines the degree of randomness of the design.

The Bayesian Biased Coin Design (BBCD) proposed under the name 'dominant biased coin

design' by Antognini and Zagoraiou (2014) is similar to ABCD. Here, the probability p_i of allocating the ith patient to the experimental group depends on the acceleration parameter $a > 0$ and the ratio $N_A(i-1)/N_B(i-1)$ where $N_A(i-1)$ and $N_B(i-1)$ are the numbers of patients in groups A and B respectively after allocating $i-1$ patients.

Wei's Urn Design (UD) consists of N draws of an urn whose composition is updated after each draw. Before allocating the first patient, the urn contains an initial number ($ini \geq 0$) of balls of different colors for each treatment. For the allocation of a patient, a ball is drawn, the color is noted, and replaced along with an additional number ($add \geq 0$) of balls of the of the opposite color.

The Generalized Biased Coin Design (GBCD) was developed by Smith (1984) to extend various designs. A biased coin is tossed for the allocation of patients, where probability of allocating the next patient to the experimental group depends on $N_A(i-1)$ and $N_B(i-1)$ as well as a balancing parameter $\rho \geq 0$.

The Hadamard Randomization (HADA) proposed by Bailey and Nelson (2003) uses the rows of a special Hadamard matrix $H \in \{0,1\}^{11\times 12}$ for the allocation of patients. Rows from H are sampled until the number of allocations reaches the planned sample size N.

Table 3 in the next chapter shows how the presented randomization procedures can be used in **randomizeR**.

2.4. Implementation

The assessment of a randomization procedure is based on a set of allocation sequences. Some of the assessment criteria, for example type-I-error or power, depend on a response, while others, e.g., correct guesses, are independent of a response. There are two different options to generate a set of allocation sequences depending on the sample size N, and two different methods to calculate the response based assessment criteria:

- **Complete or simulated reference set:** In case of $N \leq 24$, it is possible to generate the set of all possible allocation sequences $\{0,1\}^N$, assess the eligibility of an allocation sequence and, independently, calculate the associated probabilities for the allocation sequences by the randomization algorithm. This results in the *complete set* of the sequences. The function `getAllSeq` provides this functionality, see Section 3.2.3.

  In case of $N > 24$, the complete set cannot be calculated in reasonable time. Instead, a number r of allocation sequences is generated undergoing the randomization algorithm of the particular randomization procedure. The function `genSeq` provides this functionality, see Section 3.2.3. Formally, the relative frequencies of the allocation sequences can be used to estimate the true probabilities of allocation sequences. Note therefore that, in a simulation, the relative frequency of a randomization sequence is used. This results in the *simulated set* of the sequences. The simulated set can also be applied in case of smaller sample sizes, and procedures where the exact approach is not available in the literature, i.e., Block Randomization with random block constellation and Hadamard Randomization.

- **Exact or simulated response based assessment criteria:** The susceptibility of a randomization procedure to bias can be measured as the distortion of the type-I- or type-II-error probability. The *exact method* computes the distribution of the exact rejection probabilities. For each randomization sequence in the reference set, the rejection

probability of Student's t test is calculated using the knowledge on the b_i from Model 3 (see Langer 2014, Chapter 4). The *simulation method* simulates a response vector for each allocation sequence in the reference set according to Model 3 and derives a test decision of Student's t test. The type-I- or type-II-error *rate* is computed as the proportion of falsely rejected test decisions. This method was used for example by Proschan (1994). Section 3.3 shows how the exact and simulated type-I- and type-II-error probabilities can be assessed with **randomizeR**.

Any combination of the above methods can be used. For example, a simulated reference set can be used to assess the distribution of the exact rejection probabilities. The combination of simulated error rate with simulated reference set is usually used in the literature. For small sample sizes, the combination of exact reference set with distribution of exact error probabilities yields most accurate results.

All sampling algorithms use R's standard random number generator, the Mersenne-Twister (R Core Team 2016).

3. The randomizeR package

3.1. Overview

The **randomizeR** package covers two closely connected purposes: The generation of randomization sequences and the assessment of randomization procedures according to the aforementioned criteria. The previous chapter was dedicated to introducing the basic terms and literature from the field. In the present chapter, we show how **randomizeR** addresses these purposes.

The current version of **randomizeR** is based on R 3.3.0. It can be loaded in an R session via:

```
R> library("randomizeR")
```

All the main components of **randomizeR** are implemented using the S4 object oriented system.

3.2. Generating randomization sequences

There are two main purposes for the generation of randomization sequences. The first purpose is the generation of a single sequence for the allocation of patients in a clinical trial. The second purpose is the generation of multiple sequences in order to assess the properties of a randomization procedure. Both purposes can be regarded as functions that use the randomization procedure itself as basis for their behaviour. They are therefore implemented as methods that take an object representing the randomization procedure as input.

Representing randomization procedures

randomizeR implements randomization procedures as subclasses of the `randPar` class. For example, an object representing Complete Randomization with sample size $N = 10$ is generated by

```
R> N <- 10
R> (params <- crPar(N))


Object of class "crPar"

design = CR
N = 10
groups = A B
```

The function `crPar` is a so-called constructor function, namely a function that generates an object of the class `crPar` and prepares it for use. The object `param` then contains all the information about the randomization procedure. Table 3 summarizes the constructor functions for the randomization procedures described in Section 2.3. In **randomizeR** an overview over the implemented randomization procedures is shown by `?randPar`.

Generation of a single sequence

We can use the function `genSeq` to generate a single randomization sequence for a particular clinical trial. It takes an object representing a randomization procedure as input and generates a sequence at random using this procedure. For example, the following code generates a randomization sequence using Complete Randomization with sample size $N = 10$ as above:

```
R> params <- crPar(N)
R> (R <- genSeq(params))


Object of class "rCrSeq"

design = CR
seed = 808898100
N = 10
groups = A B

The sequence M:

1 B A A A B A B A A A
```

To ensure the reproducibility of the results and to enhance the reporting of the randomization procedure, the function `genSeq` saves all the information that was used for the generation of the randomization sequence in the object `R` along with the randomization sequence itself.

In order to obtain the randomization sequence stored in the object `R`, we can use the function `getRandList`:

```
R> getRandList(R)

     [,1] [,2] [,3] [,4] [,5] [,6] [,7] [,8] [,9] [,10]
[1,] "B"  "A"  "A"  "A"  "B"  "A"  "B"  "A"  "A"  "A"
```

Randomization procedure	Constructor function	Parameters
Complete Randomization (CR)	`crPar(N)`	`N` sample size
Random Allocation Rule (RAR)	`rarPar(N)`	`N` sample size
Permuted Block Randomization (PBR)	`pbrPar(bc)`	`bc` block constellation
Rand. Permuted Block Randomization (RPBR)	`rpbrPar(N, rb)`	`N` sample size<br>`rb` random block lengths
Truncated Binomial Design (TBD)	`tbdPar(bc)`	`bc` block constellation
Rand. Truncated Binomial Design (RTBD)	`rtbdPar(N, rb)`	`N` sample size<br>`rb` random block lengths
Maximal Procedure (MP)	`mpPar(N, mti)`	`N` sample size<br>`mti` max. tolerated imbalance
Big Stick Design (BSD)	`bsdPar(N, mti)`	`N` sample size<br>`mti` max. tolerated imbalance
Efrons Biased Coin Design (EBC)	`ebcPar(N, p)`	`N` sample size<br>`p` biased coin probability
Chen's Design (CHEN)	`chenPar(N, mti, p)`	`N` sample size<br>`mti` max. tolerated imbalance<br>`p` biased coin probability
Generalized Biased Coin Design (GBCD)	`gbcdPar(N, rho)`	`N` sample size<br>`rho` balance factor
Adjustable Biased Coin Design (ABCD)	`abcdPar(N, a)`	`N` sample size<br>`a` balance factor
Bayesian Biased Coin Design (BBCD)	`bbcdPar(N, a)`	`N` sample size<br>`a` balance factor
Wei's Urn Design (UD)	`udPar(N, ini, add)`	`N` sample size<br>`ini` initial urn composition<br>`add` adjustment in each step
Hadamard Randomization (HADA)	`hadaPar(N)`	`N` sample size

Table 3: Randomization procedures included in **randomizeR**.

The randomization sequence and the other information stored in the object `R` can conveniently be saved to a `.csv` file by using the `saveRand` function:

```
R> saveRand(R, file="myRandList.csv")
```

Figure 1 in Section 2.3 shows the random walk of the randomization sequence `R`. This figure can be generated using the function `plotSeq` in **randomizeR**:

```
R> plotSeq(R, plotAllSeq = T)
```

The function `genSeq` can generate randomization sequences for all randomization procedures from Table 3. The function `genSeq` has a method for each randomization procedure. For all randomization procedures, its output is an object of a class extending the class `randSeq`.

Generation of a set of sequences

Randomization procedures can be assessed based on the set of sequences they produce (see Section 2.4). **randomizeR** provides two ways to generate multiple randomization sequences from a specific procedure.

For small sample sizes $N \leq 24$, we can use the function `getAllSeq` to generate the complete set of randomization sequences. It takes an object representing a randomization procedure as input and calculates the complete set of randomization sequences of that procedure. For example, in the above case of Complete Randomization with sample size $N = 10$, we run the statement

```
R> (allSeqs <- getAllSeq(params))


Object of class "crSeq"

design = CR
N = 10
groups = A B

The first 3 of 1024 sequences of M:

1 A A A A A A A A A A
2 B A A A A A A A A A
3 A B A A A A A A A A
...
```

to get the complete set of $2^{10} = 1024$ sequences. Note that the function `getAllSeq` does not support the random block designs RPBR and RTBD or the Hadamard Randomization, because no algorithms have been established in the literature.

In those cases where the enumeration of the complete set of sequences is computationally intensive or algorithmically not feasible, the function `genSeq` can be used to generate a simulated reference set. For example for sample size $N = 50$ and Complete Randomization, a simulated reference set of size $r = 10,000$ is generated by

```
R> N <- 50
R> params <- crPar(N)
R> (randomSeqs <- genSeq(params, r = 10000))
```

```
Object of class "rCrSeq"

design = CR
seed = 1811465973
N = 50
groups = A B

The first 3 of 10000 sequences of M:

1 A   A   B   B   A   B   B   B   B   A   ...
2 A   B   A   B   A   A   B   B   A   A   ...
3 B   B   A   B   A   A   B   B   B   B   ...
...
```

The parameter `seed` can be passed to `genSeq` to ensure the reproducibility of the results.

As `genSeq` samples randomization sequences with replacement and with the true probability of occurrence of the randomization procedure represented by `params`, the result may contain duplicates. More probable sequences will occur more frequently.

randomizeR provides the function `getProb` for the computation of the true probability of occurrence randomization of sequences. `getProb` takes any object that inherits from `randSeq`:

```
R> p <- getProb(allSeqs)
R> head(data.frame(Sequences = myPaste(getRandList(allSeqs)),
+                  Probability = round(p, 6)))
```

```
   Sequences Probability
1 AAAAAAAAAA    0.000977
2 BAAAAAAAAA    0.000977
3 ABAAAAAAAA    0.000977
4 BBAAAAAAAA    0.000977
5 AABAAAAAAA    0.000977
6 BABAAAAAAA    0.000977
```

If applied to an object resulting from `getAllSeq`, the resulting probabilities will always sum up to one. In contrary, if applied to `genSeq`, the sum of the probabilities will not equal one, because typically, the set of sampled sequences is only a subset of the complete set of sequences. Note therefore that, in a simulation, the sampled frequency of a randomization sequence is used instead of its true probability of occurrence. Due to the nature of the sampling algorithms, the sampled frequencies converge to the true probabilities if the sample is large enough.

3.3. Assessing randomization procedures

Most of the assessment criteria in Section 2.2 depend on the assumtion of normally distributed responses according to (1). Assume that both treatments have equal expectation $\mu_A = \mu_B = 0$ and variances $\sigma_A = \sigma_B = 1$. Then we can use the function `normEndp` representing the normal endpoint to pass these assumtions to **randomizeR** by setting

```
R> muA <- muB <- 0
R> sigmaA <- sigmaB <- 1
R> normalEndpoint <- normEndp(mu = c(muA, muB), sigma = c(sigmaA, sigmaB))
```

The class `endpoint` provides flexibility for the extension to other endpoints. Currently, only normal endpoints are available.

randomizeR implements the criteria for the evaluation of randomization procedures as subclasses of the class `issue`. For example, an object representing the exact rejection probability in the presence of chronological bias due to linear time trend that has a strength of $\vartheta = 1$, is generated by

```
R> (cb <- chronBias(type = "linT", theta = 1, method = "exact"))


 Object of class "chronBias"

         TYPE = linT
         THETA = 1
         METHOD = exact
         ALPHA = 0.05
```

The parameter `method` indicates whether the exact distribution of the type-I-error rate should be calculated or whether the test decision should be simulated by generating responses that are influenced by the trend (`method = "sim"`). The function `chronBias` is a constructor function for objects of the class `chronBias`. The object `cb` contains all the information about the bias. Table 4 summarizes the constructor functions for all the criteria of assessment presented in Section 2.2. In **randomizeR** an overview over the implemented assessment criteria is shown by `?issues`.

Assessment of a randomization procedure

The **randomizeR** package includes the function `assess` to evaluate the behavior of a randomization procedure with respect to one or more of the criteria from Section 2.2.

For example, if we want to evaluate the behavior of the Big Stick Design with sample size $N = 12$ and imbalance tolerance $mti = 2$ with respect to chronological bias, selection bias, and power loss given a difference in group means $d = 1.796$, we may call:

```
R> N <- 12
R> mti <- 2
R> bsdSeq <- getAllSeq(bsdPar(N, mti))
```

Criterion	Constructor Function	Parameters
Chronological Bias	`chronBias(type,theta, method)`	`type` of trend<br>`theta` strength of trend<br>`method` of assessment
Selection Bias	`selBias(type,eta, method)`	`type` of guessing strategy<br>`eta` selection effect<br>`method` of assessment
Correct Guesses	`corGuess(type)`	`type` of guessing strategy
Imbalance	`imbal(type)`	`type` of imbalance
Combined Bias	`combineBias(selBias, chronBias)`	`selBias` object<br>`chronBias` object
Power loss due to imbalance	`setPower(d, method)`	`d` detectable effect<br>`method` of assessment

Table 4: Criteria for the assessment.

```
R>
R> d <- 1.796
R> sb <- selBias("CS", eta = d/4, method = "exact")
R> cb <- chronBias("linT", theta = 1/N, method = "exact")
R> pw <- setPower(d, method = "exact")
```

The function `assess` yields the true rejection probability for each criterion and each randomization sequence.

```
R> (A <-assess(bsdSeq, sb, cb, pw, endp = normalEndpoint))


Assessment of a randomization procedure

design = BSD(2)
N = 12
K = 2
groups = A B


The first 3 rows of 972 rows of D:

       Sequence Probability P(rej)(CS) P(rej)(linT) power(exact)
1 BBABABABA ...       0.004      0.045         0.05        0.789
2 BABBABABA ...       0.002      0.042         0.05        0.789
3 ABBBABABA ...       0.002      0.039         0.05        0.789
...
```

In the case of the Big Stick Design with sample size $N = 12$ and maximum tolerated imbalance $mti = 2$, there are 972 possible sequences. The first column of the assessment corresponds to the randomization sequence and the second to its probability of occurrence of the sequence. The following columns correspond to the criteria `sb`, `cb` and `pw` passed to `assess`. The notation `P(rej)(type)` refers to the probability of rejection in the presence of the given `type` of bias. The column `power(exact)` gives the exact power of each randomization sequence. Any number of assessment criteria can be passed to `assess`. For the criteria `imbal` and `corGuess` the endpoint `endp` is not relevant and can be omitted.

The `summary` of the assessment shows the important characteristics of the distribution of the allocation sequences such as mean, standard deviation, minimum, maximum and the quantiles for each criterion:

```
R> summary(A)
```

```
     P(rej)(CS) P(rej)(linT) power(exact)
mean      0.056         0.05        0.795
sd        0.013         0.00        0.006
max       0.109         0.05        0.800
min       0.034         0.05        0.789
x05       0.037         0.05        0.789
x25       0.048         0.05        0.789
x50       0.054         0.05        0.789
x75       0.062         0.05        0.800
x95       0.079         0.05        0.800
```

For example, the five percent quantile `x05` of the probability of rejection in the presence of a linear time trend `linT` is 0.042. Note that, until now, only the mean value of each criterion has been studied in the literature.

Comparison of randomization procedures

randomizeR provides the function `compare` for the comparison of several randomization procedures with respect to one of the criteria from Section 2.2. For example, assume we are in the same setting as above ($N = 12$) and want to compare the Big Stick Design, the Maximal Procedure with $mti = 2$ and the Permuted Block Randomization with block size four with respect to their susceptibility to selection bias. We can partly recycle the previous code and set the parameters for the other randomization procedures we want to compare:

```
R> mpSeq <- getAllSeq(mpPar(N, mti))
R> bc <- rep(4, N/4)
R> pbrSeq <- getAllSeq(pbrPar(bc))
```

Then the following code compares the aforementioned procedures with respect to selection bias:

```
R> (C <- compare(sb, bsdSeq, mpSeq, pbrSeq, endp = normalEndpoint))
```

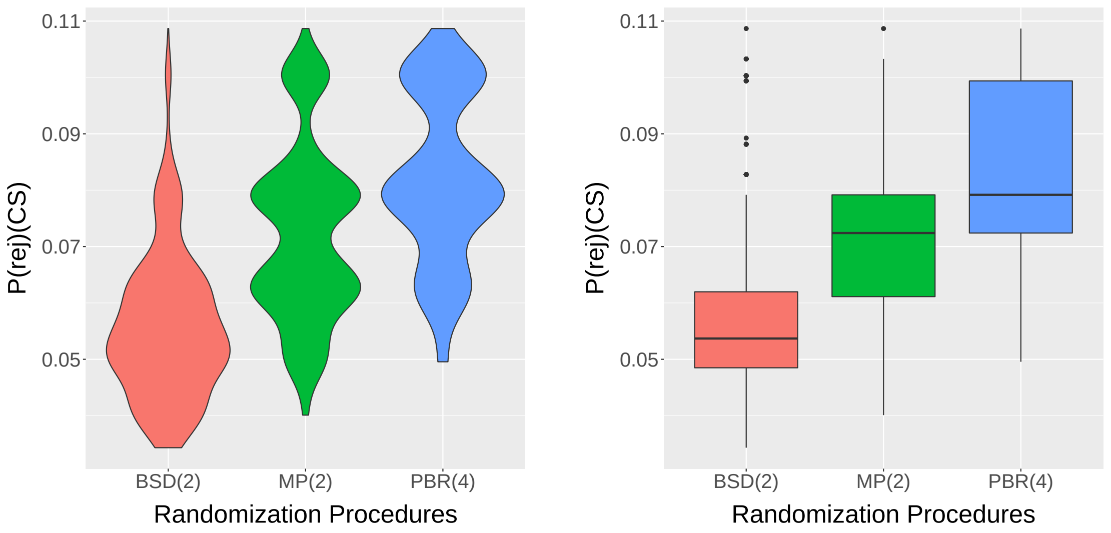

(a) Violin plot (b) Boxplot

Figure 2: Comparative visualization of the distribution of the type-I-error probability under the influence of unobserved selection bias for BSD(2), MP(2) and PBR(4).

```
Comparison for P(rej)(CS)

     BSD.2. MP.2. PBR.4.
mean  0.056 0.072  0.082
sd    0.013 0.015  0.015
max   0.109 0.109  0.109
min   0.034 0.040  0.050
x05   0.037 0.050  0.061
x25   0.048 0.061  0.072
x50   0.054 0.072  0.079
x75   0.062 0.079  0.099
x95   0.079 0.100  0.103
```

The distribution of a criterion for a particular randomization procedure can be visualized and compared between different randomization procedures via boxplot (Figure 2b) or violin plot (Figure 2a):

```
R> plot(C)
R> plot(C, y = "boxplot")
```

For the generation of boxplot and violin plot, we used the functions `geom_boxplot` and `geom_violin` of the R package **ggplot2** proposed by Wickham (2009).

4. Summary and further research

randomizeR is an R package that facilitates the generation of randomization lists for a large number of randomization procedures and makes the assessment of randomization procedures with respect to various criteria possible. The package currently implements 15 randomization procedures and six criteria for the assessment of the procedures. It assists researchers at the design stage of a clinical trial by letting the choice of a randomization procedure and the implementation of the design go hand in hand.

We are working towards extending **randomizeR** in various directions. The object oriented approach makes it easy to add new randomization procedures and assessment criteria. A unified assessment criterion may be included to uniformly judge the suitability of a randomization procedure based on various criteria. The models may be extended to other endpoints such as time-to-event data, or to more than two treatment groups. Finally, randomization tests may be implemented to enable randomization based inference independent of parametric assumptions.

Acknowledgement

This research has received funding within the IDeAl FP7 project from the European Union's Seventh Framework Programme for research, technological development and demonstration under Grant Agreement no 602552.

References

Antognini AB, Giovagnoli A (2004). "A new 'biased coin design' for the sequential allocation of two treatments." *Journal of the Royal Statistical Society: Series C (Applied Statistics)*, **53**(4), 651–664. ISSN 1467-9876. URL `http://dx.doi.org/10.1111/j.1467-9876.2004.00436.x`.

Antognini AB, Zagoraiou M (2014). "Balance and randomness in sequential clinical trials: the dominant biased coin design." *Pharmaceutical Statistics*, **13**(2), 119–127. ISSN 1539-1612. URL `http://dx.doi.org/10.1002/pst.1607`.

Armitage P (1982). "The role of randomization in clinical trials." *Statistics in Medicine*, **1**(4), 345–352. ISSN 1097-0258. URL `http://dx.doi.org/10.1002/sim.4780010412`.

Atkinson AC (2014). "Selecting a Biased-Coin Design." *Statist. Sci.*, **29**(1), 144–163. URL `http://dx.doi.org/10.1214/13-STS449`.

Bailey RA, Nelson P (2003). "Hadamard Randomization: a valid restriction of random permuted blocks." *Biometrical Journal*, **45**, 554–560.

Berger VW, Bejleri K, Agnor R (2015). "Comparing MTI randomization procedures to blocked randomization." *Statistics in Medicine*, pp. n/a–n/a. ISSN 1097-0258. URL `http://dx.doi.org/10.1002/sim.6637`.

Berger VW, Ivanova A, Knoll DM (2003). "Minimizing predictability while retaining balance through the use of less restrictive randomization procedures." *Statistics in Medicine*, **22**(19), 3017–3028. ISSN 1097-0258. URL http://dx.doi.org/10.1002/sim.1538.

Blackwell D, Hodges J (1957). "Design for the control of selection bias." *Annals of Mathematical Statistics*, **25**, 449–460.

Chalmers I (1999). "Why transition from alternation to randomisation in clinical trials was made." *BMJ*, **319**(7221), 1372. ISSN 0959-8138. URL http://www.bmj.com/content/319/7221/1372.

Chen YP (1999). "Biased coin design with imbalance tolerance." *Communications in Statistics. Stochastic Models*, **15**(5), 953–975. URL http://dx.doi.org/10.1080/15326349908807570.

Efron B (1971). "Forcing a sequential experiment to be balanced." *Biometrika*, **58**(3), 403–417. URL http://biomet.oxfordjournals.org/content/58/3/403.abstract.

Heussen N (2004). *Der Einfluss der Randomisierung in Blöcken zufälliger Länge auf die Auswertung klinischer Studien mittels Randomisationstest.* Ph.D. thesis, RWTH Aachen University.

ICH E9 (1998). "Statistical principles for clinical trials." *Current version dated 5 Februrary 1998. Last access in September 2014. Available from:* http://www.ich.org.

Kennes LN, Hilgers RD, Heussen N (2012). "Choice of the Reference Set in a Randomization Test Based on Linear Ranks in the Presence of Missing Values." *Communications in Statistics - Simulation and Computation*, **41**, 1051–1061.

Lachin JM (1988). "Statistical Properties of Randomization in Clinical Trials." *Elsevier Sdence Publishing Co., Inc.*

Langer S (2014). *The modified distribution of the t-test statistic under the influence of selection bias based on random allocation rule.* Master's thesis, RWTH Aachen.

Matts JP, McHugh RB (1978). "Analysis of accrual randomized clinical trials with balanced groups in strata." *Journal of Chronic Diseases*, **31**(12), 725 – 740. ISSN 0021-9681. URL http://www.sciencedirect.com/science/article/pii/0021968178900577.

Proschan M (1994). "Influence of selection bias on type 1 error rate under random permuted block designs." *Statistica Sinica*, **4**, 219–231.

R Core Team (2016). *R: A Language and Environment for Statistical Computing.* R Foundation for Statistical Computing, Vienna, Austria. URL https://www.R-project.org/.

Rosenberger W, Lachin J (2016). *Randomization in Clinical Trials: Theory and Practice.* Wiley Series in Probability and Statistics. Wiley. ISBN 9781118742242. URL https://books.google.de/books?id=ZJEvCgAAQBAJ.

Rosenkranz GK (2011). "The Impact of Randomization on the Analysis of Clinical Trials." *Statistics in Medicine*, **30**, 3475–3487.

Ryeznik Y, Sverdlov O, Wong WK (2015). "RARtool: A MATLAB Software Package for Designing Response-Adaptive Randomized Clinical Trials with Time-to-Event Outcomes." *Journal of Statistical Software*, **66**(1), 1–22. ISSN 1548-7660. doi:10.18637/jss.v066.i01. URL https://www.jstatsoft.org/index.php/jss/article/view/v066i01.

Smith RL (1984). "Sequential Treatment Allocation Using Biased Coin Designs." *Journal of the Royal Statistical Society. Series B (Methodological)*, **46**(3), 519–543. URL http://www.jstor.org/stable/2345691.

Snow G (2013). *blockrand: Randomization for block random clinical trials*. R package version 1.3, URL https://CRAN.R-project.org/package=blockrand.

Soares JF, Wu C (1983). "Some Restricted randomization rules in sequential designs." *Communications in Statistics - Theory and Methods*, **12**(17), 2017–2034. http://dx.doi.org/10.1080/03610928308828586, URL http://dx.doi.org/10.1080/03610928308828586.

Tamm M, Cramer E, Kennes LN, Heussen N (2012). "Influence of selection bias on the test decision - a simulation study." *Methods of Information in Medicine*, **51**, 138–143.

Tamm M, Hilgers RD (2014). "Chronolgical bias in randomized clinical trials under different types of unobserved time trends." *Meth. Inf. Med.*, **53**(6), 501–510.

Uschner D, Schindler D, Heussen N, Hilgers RD (2016). "randomizeR: An R Package for the Assessment and Implementation of Randomization in Clinical Trials." *submitted to the Journal of Statistical Software*.

Wickham H (2009). *ggplot2: Elegant Graphics for Data Analysis*. Springer-Verlag New York. ISBN 978-0-387-98140-6. URL http://ggplot2.org.

Affiliation:

Diane Uschner
Department of Medical Statistics
RWTH Aachen University
Pauwelsstraße 30
52068 Aachen, Germany
E-mail: duschner@ukaachen.de
URL: http://www.ideal.rwth-aachen.de/
