# Supplementary material for: Randomization in survival studies: An evaluation method that takes into account selection and chronological bias
Source: PLoS One. 2019 Jun 3;14(6):e0217946. doi: 10.1371/journal.pone.0217946 (PMC6546249; doi:10.1371/journal.pone.0217946)
Supplement: S3 File — Source code of the randomizeR package version 2.0. (GZ) [file pone.0217946.s003.gz › randomizeR/inst/doc/comparison-example.pdf]

Tutorial: comparing randomization procedures with randomizeR.

Diane Uschner

May 24, 2019

1 Introduction

randomizeR is a user-friendly package that allows the user to assess and compare randomization procedures according to issues. This tutorial focuses on the comparison of randomization procedures.

To install randomizeR from CRAN, run

```
install.packages("randomizeR")
```

in your R command line.

2 Main working example

Assume we are in the planning stage of a two-armed clinical trial with $N = 24$ patients. Assume further that we want to choose a randomization procedure such that the potential for selection bias is minimized. randomizeR can help us choose a suitable randomization procedure by comparing various randomization procedures subject to their susceptibility to selection bias. In order to use the functionality provided by randomizeR, we have to load it into the library:

```
library(randomizeR)
```

Blackwell and Hodges 1957 proposed the expected proportion of correct guesses as a measure for the susceptibility to selection bias. They also showed that the "'convergence strategy"' is optimal if the randomization procedure forces terminal balance. randomizeR represents the expected proportion of correct guesses with convergence strategy ("'CS"') as follows:

```
cg <- corGuess("CS")
cg
```

```
##
##  Object of class "corGuess"
##
##   TYPE = CS
```

Because we want to reuse this representation of the correct guesses, we assigned it to the variable `cg`. `randomizeR` supports five different criteria (aka. `issues`) for the comparison of randomization procedures. The help page

```
?issues
```

provides an overview over all implemented criteria.

Now assume that we are interested in a comparison between the Permuted Block Randomization with block length four (PBR(4)), Random Allocation Rule (RAR) and the Big Stick Design with imbalance tolerance two (BSD(2)). (see Rosenberger and Lachin 2002) Again, we can represent these randomization procedures in `randomizeR` easily. Starting with RAR, we get

```
rar <- rarPar(24)
rar

##
## Object of class "rarPar"
##
## design = RAR
## N = 24
## groups = A B
```

For PBR(4), we need to define the *block constellation* `bc`, i.e. the sequence of blocks that are forced to be balanced; and then pass it to `pbrPar`.

```
bc <- rep(4, 24/4)
pbr <- pbrPar(bc)
pbr

##
## Object of class "pbrPar"
##
## design = PBR(4)
## bc = 4 4 4 4 4 4
## N = 24
## groups = A B
```

BSD(2) depends on the total sample size and the maximum tolerated imbalance:

```
bsd <- bsdPar(24, 2)
bsd

##
## Object of class "bsdPar"
##
## design = BSD(2)
## mti = 2
## N = 24
## groups = A B
```

The objects **rar**, **pbr** and **bsd** now represent the randomization procedures RAR, PBR(4) and BSD(2) for $N = 24$ and we can use them in our calculations. If you are interested in more randomzation procedures, you can similarly choose any of the ten randomization procedures supported by **randomizeR**. The help page

```
?randPar
```

provides an overview over all randomization procedures implemented.

randomizeR pursues a sequence based approach. That means that the randomization procedures are compared based on the randomization sequences they produce along with their probability of occurrence. Naturally, the sampling algorithms of **randomizeR** implement the randomization procedures such that the theoretical probabilities of occurence equal the sampled relative frequencies.

For the comparison of **rar**, **pbr** and **bsd** we thus need to generate sequences. This is fairly easy in **randomizeR**

```
rarS <- genSeq(rar, r = 1000, seed = 123)
pbrS <- genSeq(pbr, r = 1000, seed = 124)
bsdS <- genSeq(bsd, r = 1000, seed = 125)
```

The resulting objects include 1000 random sequences from x for $x \in \{$**rar**, **pbr**, **bsd**$\}$, along with the information of the randomization procedure.

```
bsdS

##
## Object of class "rBsdSeq"
##
## design = BSD(2)
## seed = 125
## N = 24
## groups = A B
## mti = 2
##
```

```
## The first 3 of 1000 sequences of M:
##
## 1 B   A   A   A   B   B   B   B   A   A   ...
## 2 A   B   B   A   B   A   B   A   A   A   ...
## 3 A   A   B   B   B   B   A   A   B   B   ...
## ...
```

That way the information about how the sequences have been generated can never get lost. The sequences themselves can be accessed via

```
getRandList(bsdS)
```

Now we are ready to actually compare the randomization procedures. This is easy, because we can recycle all the objects that we have generated already. In `randomizeR`, we simply call `compare` and pass all the arguments to it:

```
C <- compare(cg, rarS, pbrS, bsdS)
C

##
## Comparison for propCG(CS)
##
##        RAR PBR.4. BSD.2.
## mean 0.610  0.708  0.616
## sd   0.047  0.024  0.043
## max  0.750  0.750  0.729
## min  0.521  0.625  0.479
## x05  0.542  0.667  0.542
## x25  0.583  0.688  0.583
## x50  0.604  0.708  0.625
## x75  0.646  0.729  0.646
## x95  0.688  0.750  0.688
```

The first argument must be an `issue`, and the following arguments must be of class `randSeq`, i.e. representations of randomization sequences.

From the table we can see that the maximum extent for selection bias is equal for RAR and PBR, while it is slightly lower for BSD. Concerning the mean, RAR and BSD keep nearly the same level. The minimum however is higher for RAR, indicating that all RAR sequences have inflated type-I-error probability in case a selection bias is present. To make things even more accessible, `randomizeR` provides a function for visualizing this comparison:

```
plot(C)
```

Figure 1 shows the result of this call. The target value of the expected proportion of correct guesses is 0.5. Figure 1 shows that BSD manages to keep

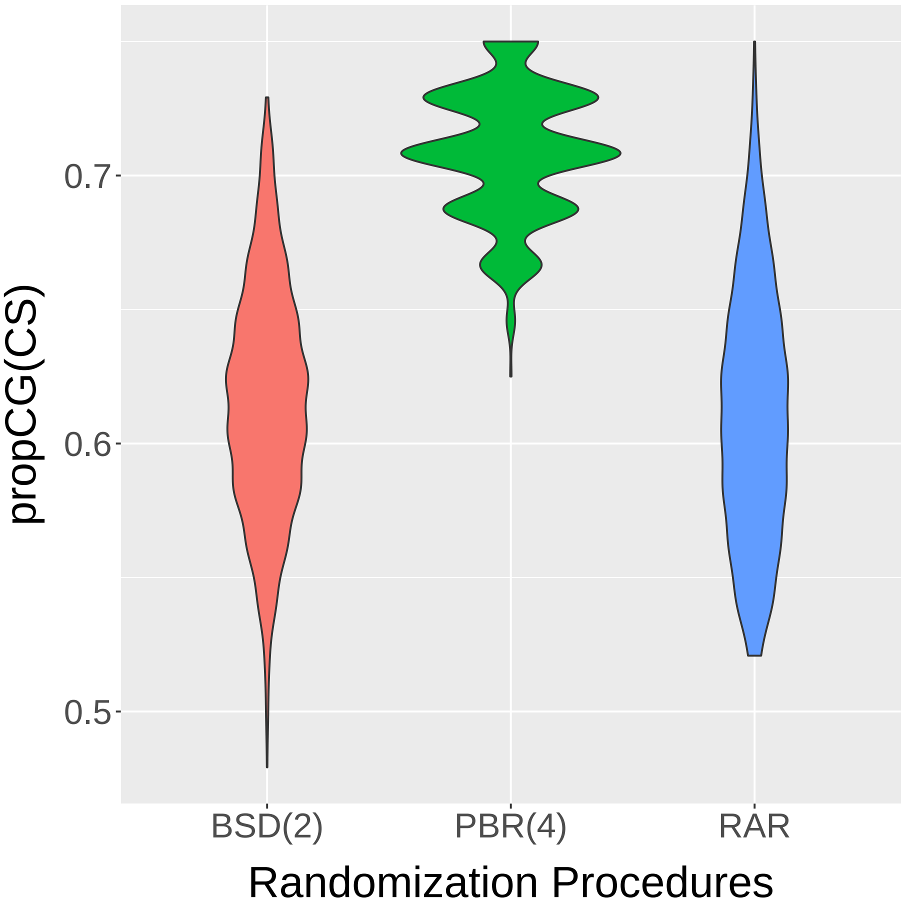

Figure 1: Comparison of randomization procedures

this target value for some sequences, and extends to approximately the same maximal value as RAR. PBR attains a higher extent of selection bias, yielding it inferior to BSD and RAR. It can thus be concluded that BSD manages selection bias best of the three investigated procedures.

3 Conclusion

`randomizeR` makes the comparison of different randomization procedures according to one criterion easy. Furthermore, it comes with an extensive number of randomization procedures and incorporates all relevant demands on randomization procedures. The user can thus choose a tailored randomization procedure on a scientifically sound basis.

References

[BH57] D. Blackwell and J. Hodges. "Design for the control of selection bias". In: *Annals of Mathematical Statistics* 25 (1957), pp. 449–460.

[RL02] W. F. Rosenberger and J. M. Lachin. *Randomization in Clinical Trials-Theory and Practice.* Wiley Series in probability and statistics, 2002. URL: `http://books.google.de/books?id=Wy0hy4DPEPQC`.
