# Supplementary material for: Randomization in survival studies: An evaluation method that takes into account selection and chronological bias
Source: PLoS One. 2019 Jun 3;14(6):e0217946. doi: 10.1371/journal.pone.0217946 (PMC6546249; doi:10.1371/journal.pone.0217946)

Tutorial: Interplay between functions related to desirability indices within `randomizeR`.

David Schindler, Diane Uschner, Thi Mui Pham

May 24, 2019

1 Introduction

`randomizeR` is a user-friendly package that allows the user to assess and compare randomization procedures according to certain criteria (called issues in this package). This tutorial focuses on the comparison of randomization sequences and procedures on the basis of desirability functions. In this context, there are certain criteria and requirements given which have to be met by a randomization procedure. Desirability scores are a kind of linked optimization criteria that establish the connection between the given requirements and therby allow a comparison between them.

Currently, `randomizeR` encompasses the class of desirability functions introduced by Derringer and Suich (1980) and corresponding functions to evaluate and compare randomization sequences which have been assessed on the basis of desirability indices of specific issues.

For each value x of the corresponding criterion, a desirability function d assigns numbers betweens 0 and 1 to the possible values of x with $d(x) = 0$ representing a completely undesirable value and $d(x) = 1$ representing a completely desirable or ideal value. Therefore, values with different domains are mapped to a common scale $[0, 1]$, making them comparable.

The Derringer-Suich approach expects a lower and upper specified border and an optimal desired value called the target value. There are three different kinds of desirability functions: The (left-)one-sided Derringer-Suich desirability function, the (right-)one-sided Derringer-Suich desirability function and the two-sided Derringer-Suich desirability function. All three options are supported within the `randomizeR` package. Whenever an argument x is smaller/greater than a lower/upper specified border it is mapped to zero by the desirability function. If the value of x corresponds (or even exceeds) its target value the desirability function $d(x)$ is one. The upper specified border is called upper specification limit (USL) and the lower specified border is called lower specification limit (LSL).

The functions realizing the idea described above are given by

```
derFunc
getDesScores
evaluate
probUnDes
plotDes
plotEv
```

In order to use the functions mentioned above, we have to load the `randomizeR` package into the library:

```
library(randomizeR)
```

2 Working example

Assume we would like to assess the full set of randomization sequences of sample size 4 generated by the Random Allocation Rule and based on specific issues. The behaviour of randomization sequences with respect to these issues can be evaluated by the `assess` function:

```
sequences <- getAllSeq(rarPar(4))
issue1 <- corGuess("CS")
issue2 <- chronBias("linT", 0.25, "exact")
endp <- normEndp(mu = c(0,0), sigma = c(1,1))
A <- assess(sequences, issue1, issue2, endp = endp)
A

##
## Assessment of a randomization procedure
##
## design = RAR
## N = 4
## K = 2
## groups = A B
##
##
## The first 3 rows of 6 rows of D:
##
##   Sequence Probability propCG(CS) P(rej)(linT)
## 1     BBAA       0.167      0.625        0.051
## 2     BABA       0.167      0.750        0.050
## 3     ABBA       0.167      0.750        0.049
## ...
```

However, the assessment output of different issues usually have different domains making the comparison difficult. By using desirability functions the

assessment output is scaled to $[0, 1]$. In this package `derFunc` represents both the one and two-sided desirability functions according to Derringer and Suich.

```
d1 <- derFunc(0.5, 0.75, 1)
d1

##
## Object of class "derringerRs"
##
## desirability function = derringerRs(0.5, 0.75, 1)
## SLs = 0.75
## b = 1
## TV = 0.5

d2 <- derFunc(0.05, 0.1, 1)
d2

##
## Object of class "derringerRs"
##
## desirability function = derringerRs(0.05, 0.1, 1)
## SLs = 0.1
## b = 1
## TV = 0.05
```

The desirability functions defined above are right- and left-sided respectively. By applying the `getDesScores` function to the assessment output together with the specified desirability functions the behaviour of randomization sequences is evaluated and scaled to $[0, 1]$. By combining the individual desirabilities values of the issues using the geometric mean, the *overall desirability of a given randomization sequence* is computed.

```
D1 <- getDesScores(A, d1, d2)
print(D1$D, digits = 3)

##   Sequence Probability d(propCG(CS)) d(P(rej)(linT)) geometricMean
## 1     BBAA       0.167           0.5           0.977         0.699
## 2     BABA       0.167           0.0           1.000         0.000
## 3     ABBA       0.167           0.0           1.000         0.000
## 4     BAAB       0.167           0.0           1.000         0.000
## 5     ABAB       0.167           0.0           1.000         0.000
## 6     AABB       0.167           0.5           0.977         0.699
```

Notice that regarding the expected proportion of correct guesses and with respect to the right-sided desirability function `d1` only two of six sequences are not mapped to zero. Thus, the values of correct guesses of sequences $2 - 5$

are undesirable. On the other hand, regarding linear time trend (chronological bias) and with respect to the desirability function `d2` the respective sequences are completely desirable as they are all mapped to 1. The last column displays the overall desirability. Notice that if the value of any considered issue is completely undesirable, then the corresponding overall desirability is zero.

The `getDesScore` function also allows specifying weights in order to adjust the computation of the overall desirability, i.e. the geometric mean. If, like in the case above, the weights are not specified, then the issues are equally weighted.

```
getDesScores(A, d1, d2, weights = c(5/6, 1/6))

##
## Object of class "desScores"
##
## design = RAR
## N = 4
## K = 2
## groups = A B
## desFuncs = derringerRs(0.5, 0.75, 1) derringerRs(0.05, 0.1, 1)
## weights = 0.833 0.167
##
##
## The first 3 rows of 6 rows of D:
##
##   Sequence Probability d(propCG(CS)) d(P(rej)(linT)) geometricMean
## 1     BBAA       0.167           0.5           0.977         0.559
## 2     BABA       0.167           0.0           1.000         0.000
## 3     ABBA       0.167           0.0           1.000         0.000
## ...
```

By applying the `summary` function to this object it summarizes the results by means of statistics like mean, median, min, max etc. Therefore, the third column of the `summary` output represents the *overall desirability of a given randomization procedure.*

```
summary(D1)

##      d(propCG(CS)) d(P(rej)(linT)) geometricMean
## mean         0.167           0.992         0.233
## sd           0.258           0.012         0.361
## max          0.500           1.000         0.699
## min          0.000           0.977         0.000
## x05          0.000           0.977         0.000
## x25          0.000           0.977         0.000
## x50          0.000           1.000         0.000
```

```
## x75            0.500          1.000          0.699
## x95            0.500          1.000          0.699
```

The `randomizeR` package also offers the option of visualization by plotting an `desScore` object. Moreover, by specifying the variable `quantiles`, the user can decide whether the quantiles should be depicted in the plot or not. This alllows a visualization of the summarized results (see above).

```
plotDes(D1, quantiles = TRUE)
```

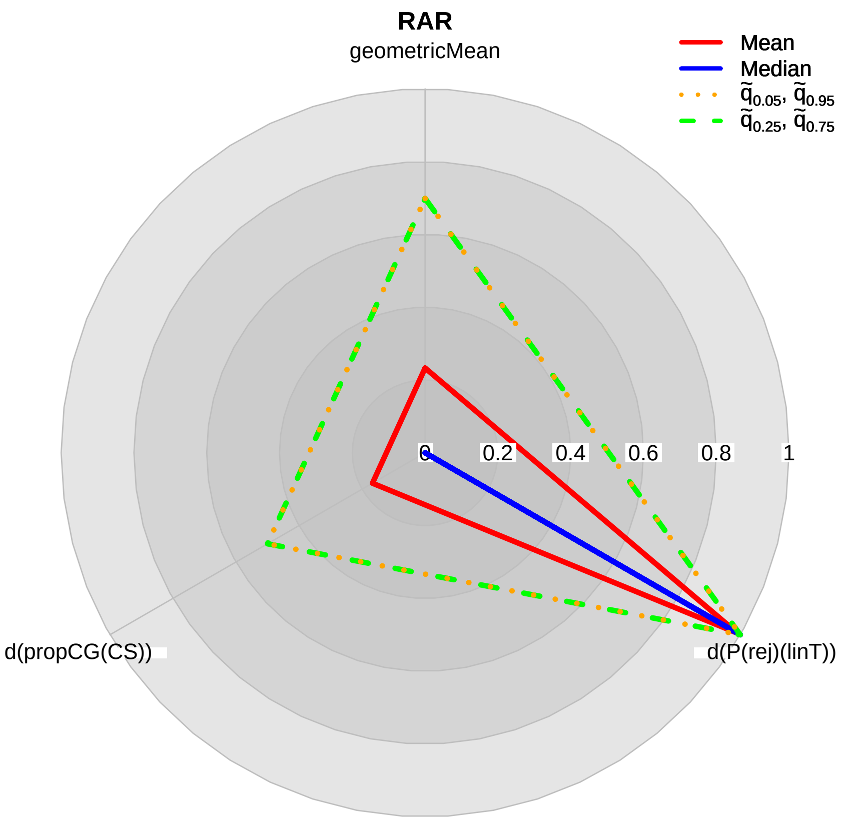

Now, suppose we would like to perform a comparison of sequences from different randomization procedures. Then this can be done by using the `evaluate` function. It expects a number of objects resulting from `getDesScores` and summarizes their results according to a given statistic. The user can choose between `mean, median, min` or `max`. If no statistic is specified then it is set to `mean`.

```r
issue1 <- corGuess("CS")
issue2 <- chronBias(type = "linT", theta = 1/4, method = "exact")
RAR <- getAllSeq(rarPar(4))
BSD <- getAllSeq(bsdPar(4, mti = 2))
A1 <- assess(RAR, issue1, issue2, endp = normEndp(c(0,0), c(1,1)))
A2 <- assess(BSD, issue1, issue2, endp = normEndp(c(0,0), c(1,1)))

d1 <- derFunc(TV = 0.5, 0.75, 2)
d2 <- derFunc(0.05, c(0, 0.1), c(1, 1))
DesScore <- getDesScores(A1, d1, d2, weights = c(5/6, 1/6))
DesScore2 <- getDesScores(A2, d1, d2, weights = c(5/6, 1/6))

evaluate(DesScore, DesScore2)

##
## Object of class "evaluation"
##
## desFuncs = derringerRs(0.5, 0.75, 2) derringerTs(0.05, 0, 0.1, 1, 1)
## weights = 0.833 0.167
## statistic = mean
##
##   RandProc d(propCG(CS)) d(P(rej)(linT)) geometricMean
## 1      RAR         0.083           0.985         0.105
## 2   BSD(2)         0.562           0.985         0.577
```

Looking at the corresponding output of `getDesScores`, we recognize that the desirability functions map a lot of values to zero, i.e. a lot of undesired sequences are among the assessed ones:

```r
print(DesScore$D, digits = 3)

##   Sequence Probability d(propCG(CS)) d(P(rej)(linT)) geometricMean
## 1     BBAA       0.167          0.25           0.977         0.314
## 2     BABA       0.167          0.00           0.994         0.000
## 3     ABBA       0.167          0.00           0.984         0.000
## 4     BAAB       0.167          0.00           0.984         0.000
## 5     ABAB       0.167          0.00           0.994         0.000
## 6     AABB       0.167          0.25           0.977         0.314

print(DesScore2$D, digits = 3)

##    Sequence Probability d(propCG(CS)) d(P(rej)(linT)) geometricMean
## 1      BAAA      0.0625          1.00           0.987         0.998
## 2      ABAA      0.0625          1.00           0.987         0.998
## 3      BBAA      0.1250          0.25           0.977         0.314
## 4      AABA      0.1250          1.00           0.987         0.998
```

```
## 5      BABA     0.0625          0.00            0.994          0.000
## 6      ABBA     0.0625          0.00            0.984          0.000
## 7      BAAB     0.0625          0.00            0.984          0.000
## 8      ABAB     0.0625          0.00            0.994          0.000
## 9      BBAB     0.1250          1.00            0.987          0.998
## 10     AABB     0.1250          0.25            0.977          0.314
## 11     BABB     0.0625          1.00            0.987          0.998
## 12     ABBB     0.0625          1.00            0.987          0.998
```

This observation raises the question which randomzation procedure produces more undesired randomization sequences with respect to certain issues and desirability functions? The function `probUnDes` computes the probability of having desirabilty scores of zero for each desirability function applied to an issue:

```
probUnDes(DesScore)

##
## Object of class "probUnDesirable"
##
## design = RAR
## N = 4
## K = 2
## desFuncs = derringerRs(0.5, 0.75, 2) derringerTs(0.05, 0, 0.1, 1, 1)
## weights = 0.833 0.167
##
##   P(d(propCG(CS))=0) P(d(P(rej)(linT))=0) P(geometricMean=0)
## 1              0.667                    0              0.667

probUnDes(DesScore2)

##
## Object of class "probUnDesirable"
##
## design = BSD(2)
## N = 4
## K = 2
## desFuncs = derringerRs(0.5, 0.75, 2) derringerTs(0.05, 0, 0.1, 1, 1)
## weights = 0.833 0.167
##
##   P(d(propCG(CS))=0) P(d(P(rej)(linT))=0) P(geometricMean=0)
## 1               0.25                    0               0.25
```

Thus, the probability of having undesired sequences is more than twice as high for the Random Allocation Rule than for the Big Stick design. A graphical method of displaying the comparison of randomization procedures is given by the `plotEv` function. It can be applied to an `evaluation` object which results from the `evaluate` function.

```
plotEv(evaluate(DesScore, DesScore2))
```

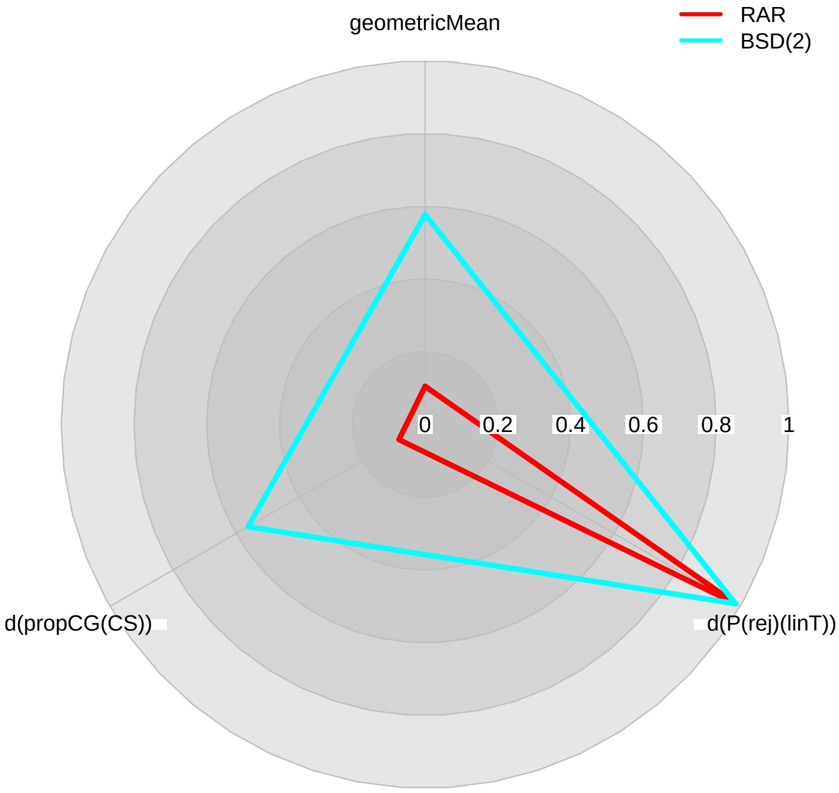

Supplement: S3 File — Source code of the randomizeR package version 2.0. (GZ) [file pone.0217946.s003.gz › randomizeR/inst/doc/desirability-example.pdf]
