# Supplementary material for: Randomization in survival studies: An evaluation method that takes into account selection and chronological bias
Source: PLoS One. 2019 Jun 3;14(6):e0217946. doi: 10.1371/journal.pone.0217946 (PMC6546249; doi:10.1371/journal.pone.0217946)
Supplement: S3 File — Source code of the randomizeR package version 2.0. (GZ) [file pone.0217946.s003.gz › randomizeR/inst/doc/randomizeR-refCard.pdf]

randomizeR 1.0 Quick Reference Guide

Diane Uschner, David Schindler, Thi Mui Pham

1 Randomization Procedures (RP)

`rarPar(N)`	Random Allocation rule with `N` patients
`crPar(N)`	Complete Randomization with `N` patients
`pbrPar(bc)`, `rpbr(bc)`	(randomized) Permuted Block Design
`ebcPar(N, p)`	Efron's Biased Coin Design with `N` patients and success probability `p`
`mpPar(N, mti)`	Maximal Procedure with `N` patients and maximum tolerated imbalance `mti`
`bsdPar(N, mti)`	Big Stick design with `N` patients and maximum tolerated imbalance `mti`
`udPar(N, ini, add)`	Wei's urn design with an initial urn composition of `ini` balls and in each step `add` balls are added
⋮	
`createParam(method, N,...)`	Creates object that represents the chosen RP
`genSeq(obj, r, seed)`	Generates random sequences
`getAllSeq(obj)`	Output of all randomization sequences for the given RP
`getProb(obj)`	Calculates theoretical probability for observed randomization sequences
`getRandList(obj)`	Get the randomization list coded in its groups
`saveRand(obj)`	Saves the generated randomization sequence and its input parameters

2 Assessment of a Randomization Procedure

Assess one randomization procedure according to several specified issues

`assess(...)`	Evaluates the behaviour of randomization sequences with respect to certain issues
`summary(assess(...))`	Summary of assessessments (for each issue) of one RP

3 Issues

Summarizes the criteria for the assessment of randomization

`selBias(type, eta, method, alpha)`	Issue of selection bias in a clinical trial with magnitude of selection bias `eta`
`chronBias(type, theta, method, alpha)`	Issue of chronological bias in a clinical trial with factor of time trend `theta`
`setPower(d, method, alpha)`	Expected power of the individual randomization sequences with effect size `d`
`normEndp(mu, sigma)`	Represents normally distributed endpoints in clinical trials (in conjunction with `assess` function and issues mentioned above)

For the parameter `method` there are two possible models supported:

`method = "sim"`	the object represents the simulated type-I-error rate given the significance level `alpha`
`method = "exact"`	the object represents the exact type-I-error probability given the significance level `alpha`
`imbal(type)`	Imbalance of the treatment assignments of patients in clinical trial with parameter `type` that represents the different kinds of imbalance, e.g. final imbalance, maximal attained imbalance
`corGuess(type)`	Expected number of correct guesses of randomization sequences with strategy parameter `type`, i.e. `type = "CS"` or `type = "DS"`

4 Comparison of Randomization procedures

Compare several randomization procedures according to one issue

`compare(issue,...)`	Compares randomization procedures based on a specified issue
`plot(compare(...))`	Creates a box- or violinplot of an object of the class `comparison`
